# Optical and theoretical study of strand recognition by nucleic acid probes

**DOI:** 10.1038/s42004-020-00362-5

**Published:** 2020-08-11

**Authors:** Ivana Domljanovic, Maria Taskova, Pâmella Miranda, Gerald Weber, Kira Astakhova

**Affiliations:** 1 Department of Chemistry, Technical University of Denmark, 206-207 Kongens, 2800 Lyngby, Denmark; 2 Department of Oncology, Microbiology and Immunology, Faculty of Science and Medicine, University of Fribourg, Per 17, Chemin du Musée 18, CH-1700 Fribourg, Switzerland; 3 Departamento de Física, Universidade Federal de Minas Gerais, Belo Horizonte-MG, Brazil; 4 Programa Interunidades de Pós-Graduação em Bioinformática, Universidade Federal de Minas Gerais, Belo Horizonte-MG, Brazil

**Keywords:** Biophysical chemistry, Nucleic acids

## Abstract

Detection of nucleic acids is crucial to the study of their basic properties and consequently to applying this knowledge to the determination of pathologies such as cancer. In this work, our goal is to determine new trends for creating diagnostic tools for cancer driver mutations. Herein, we study a library of natural and modified oligonucleotide duplexes by a combination of optical and theoretical methods. We report a profound effect of additives on the duplexes, including nucleic acids as an active crowder. Unpredictably and inconsistent with DNA+LNA/RNA duplexes, locked nucleic acids contribute poorly to mismatch discrimination in the DNA+LNA/DNA duplexes. We develop a theoretical framework that explains poor mismatch discrimination in *KRAS* oncogene. We implement our findings in a bead-bait genotyping assay to detect mutated human cancer RNA. The performance of rationally designed probes in this assay is superior to the LNA-primer polymerase chain reaction, and it agrees with sequencing data.

## Abstract (web summary)

Nucleic acid probes that bind to cancer-related genes have potential as diagnostic tools. Here parameters governing the binding of locked nucleic acid probes to three oncogenes, including effects of molecular crowding and polyelectrolytes, are characterized and used to inform the design of a bead-bait RNA genotyping assay.

## Introduction

Nucleic acid interactions play a key role in coordinating cellular functions including the encoding, transmitting, and expression of genes^1,2^. Several research and diagnostic tools rely on the ability of specific oligonucleotide probes to bind and sense their complementary target, including sequencing and the polymerase-chain reaction (PCR)^3,4^.

A particularly relevant family of targets for genomic research and clinical diagnostics are human oncogenes^5^. Mutations in oncogenes are one of the factors that can initiate cancer and contribute to its progression. Thus, single nucleotide polymorphisms (SNPs) in the oncogenes *BRAF, KRAS*, and *EGFR* are known to induce solid tumours, and they also lead to chemotherapy agent resistance^6^. Very recently, it was shown that *EGFR* mutations predict hyper-activation of tumorigenesis as a response to immunotherapy^7^. Cancer diagnostics is, therefore, a crucial task both in research and in clinical work.

Today, oncogene genotyping fully relies on synthetic oligonucleotide primers that target a specific region in cancer nucleic acids^7^. Both cancer DNA and RNA need to be targeted, although SNPs in RNA are more relevant in the direct studies of a tumour in cells and in vivo^8^.

Effective detection of SNPs in cancer DNA and RNA is a challenging task, given that only 0.1–0.0001% of the gene is mutated while the rest remains to be a wild-type^6^. SNP-specific probes largely benefit from introducing chemical modifications that improve binding affinity to a complementary target. One particular example of such a modification is locked nucleic acids (LNA)^9^. By means of an altered geometry of a sugar pocket, LNA impacts the overall 3D structure of the DNA:DNA and especially of DNA:RNA duplexes. This leads to structural reorganization and high sensitivity of LNA binding to the presence of a mismatch^10^.

The binding affinity of DNA probes is believed to be predictable, although most studies rely on measuring thermal denaturation in pure phosphate or other aqueous buffers^11,12^. Macromolecules added to the solution result in crowding that gives a rise to an “excluded volume effect”^13^. This effect may cause changes in the hydrodynamic volume, conformation, stability, structure, folding and shape of the biomolecules, and changes in association states, i.e. phase separation and biomolecular interactions^14–16^. To fully interpret the “excluded volume effect”, the influence of an inner crowder should be taken into account^17^.

The additive reagents might interact with nucleic acids as well^18^. This further complicates the fate of the synthetic probes in the biological environment. To account for this, simulations of a crowded intracellular environment have been proposed in test systems with additional reagents^19^. Polyethylene glycol (PEG) and cell lysate are among the most often applied crowders for in vitro studies^11^. The efficiency of test systems with crowding depends on the ratio between hydrodynamic dimensions of a crowder and of a test molecule^20^. However, not all additives are effective in the same way^16^. Surface charge, site-specific interactions, and intricate surface topology are factors that make additive agents complex to study^16,21,22^.

Fluorometry is a powerful technique for studies on nucleic acid hybridization. It has been actively translated into biotechnology (PCR, sequencing, microarray) and clinical work (patient diagnostics and follow up for infectious diseases, cancer, etc.)^23^. In the context of nucleic acid analyses, fluorescence relies on the properties of fluorescent dyes that are either covalently or non-covalently attached to the probe(s). When a fluorescent molecule interacts with its DNA/RNA target, it changes its absorbance and/or fluorescence properties (such as quantum yield, lifetime, or fluorescence intensity)^23^. The change in optical properties can be used as a read out for assessing the effect of the environment on the probe, enabling qualitative and quantitative measurements.

Some fluorophores interact more actively with DNA and RNA than others. Nuclear magnetic resonance (NMR) studies of dsDNA with covalently attached cyanine3 (Cy3) and cyanine5 (Cy5) dyes indicated that a dye positioned at the end of a duplex has a capping configuration that is similar to a configuration of a base pair^24^. This should increase fluorescence intensity relative to the free dye due to an arrangement that could restrict the rate of *cis*–*trans* isomerization of the dye^24^. Moreover, hybridization of two complementary strands labelled with dyes with overlapping absorbance and fluorescence properties often results in Förster resonance energy transfer (FRET)^23^. FRET is a critical tool to determine distance, configurational change, and interactions in vitro and in vivo^25^. A study that reports on crowding and FRET states that FRET probes experience compaction in a crowded solution due to smaller donor−acceptor distance and better energy-transfer efficiency^25^. The ultimate conditions for this model are through weak interactions between crowding agents, the type of FRET sensor, and steric hindrance due to the excluded volume of the crowding agents^25^.

**Fig. 1:**
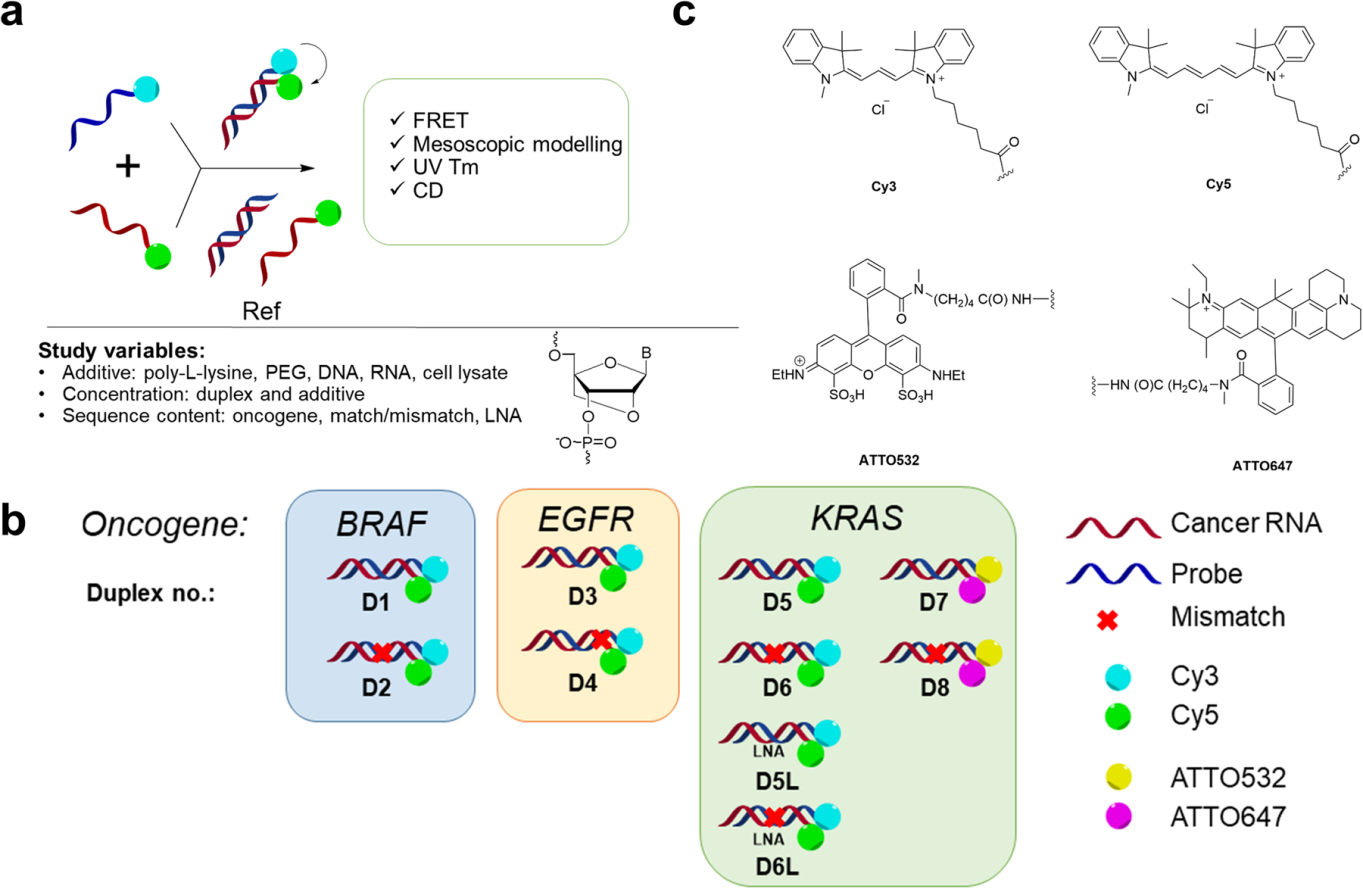
General summary of the study. **a** Representation of the assays conducted (FRET, mesoscopic modelling, UV Tm, and CD) and sample variables: additives, duplex and additive concentrations and oligonucleotide sequence content. PEG = polyethylene glycol 2000. Chemical structure of locked nucleic acid monomer, LNA, with B standing for nucleobase **b** Illustration of probes with RNA targets organized in three groups based on the targeted oncogene: *BRAF*, *KRAS*, and *EGFR*, **c** Chemical structures of FRET pairs.

Herein, we hypothesize that target recognition by synthetic oligonucleotide probes crucially depends on their chemical composition, concentration, and ratio with the additive reagents. Our objective is to design and evaluate hybridization properties of multiple model helices containing three cancer-related probes, *BRAF*, *KRAS*, and *EGFR*, bound to complementary and mismatched RNA. Ten DNA/RNA duplexes and controls are covalently conjugated with Cy3/Cy5, or ATTO532/ATTO647 FRET pairs at terminal positions (Fig. 1). We conduct detailed studies of these systems by FRET, UV thermal denaturation, circular dichroism (CD), and mesoscopic modelling at a duplex concentration range of 25–500 nM. Diverse additives and several duplex to additive ratios are included in the study. For instance, PEG, poly-l-lysine, cell lysate, genomic DNA, leukaemia cell mRNA, and short synthetic DNA duplex (Fig. 1d) are studied. Our data reported herein provides valuable clues on the optimal probe design to achieve high target-binding affinity, specificity, and sensing properties.

## Results

### Sequence design

The test systems used in this study are shown schematically in Fig. 1b; the sequences of the duplexes are given in Table 1 and in the Supplementary Table 5. Duplexes in the main group (abbreviated DR1–DR8) contained pairs of fully matched (mutant DNA probe: mutant target RNA) and mismatched (mutant DNA probe:wild type target RNA) variants. DNA mutation probes were designed to target three mutated human oncogenes: *BRAF* V600E (DR1, DR2), *EGFR* L858R (DR3, DR4), and *KRAS* G12D (DR5–DR8). Mismatched nucleotides in the corresponding duplexes are shown in Table 1, column mismatch, and within the sequences.

**Table 1:** Representation of the duplexes used in this study^a^.

Seq. ID	Mismatch	Sequences, probe: target	Oncogene
*Duplex type: DNA:RNA and DNA* + *LNA:RNA*			
DR1	–	5′-/Cy3/TAG CTA CAG **A**GA AAT CTC GAT 3′-/Cy5/rArUrC rGrArU rGrUrC **rU**rCrU rUrUrA rGrArG rCrUrA	*BRAF V600E*
DR2	dArA	5′-/Cy3/TAG CTA CAG **A**GA AAT CTC GAT 3′-/Cy5/rArUrC rGrArU rGrUrC **rA**rCrU rUrUrA rGrArG rCrUrA	*BRAF wt*
DR3	–	5′-/Cy3/**A**CT GTA CAT GAG AAA CTT TTT CTC 3′-/Cy5/**rU**rGrA rCrArU rGrUrA rCrUrC rUrUrU rGrArA rArArA rGrArG	*EGFR L858R*
DR4	dArC	5′-/Cy3/**A**CT GTA CAT GAG AAA CTT TTT CTC 3′-/Cy5/**rC**rGrA rCrArU rGrUrA rCrUrC rUrUrU rGrArA rArArA rGrArG	*EGFR wt*
DR5,DR5L	–	5′-/Cy3/GTT GGA GCT + G + **A** + T GGC GTA GGC 3′-/Cy5/rCrArA rCrCrU rCrGrA rC**rU**rA rCrCrG rCrArU rCrCrG	*KRAS G12D*
DR6 DR6L	dArC +ArC	5′-/Cy3/GTT GGA GCT + G + **A** + T GGC GTA GGC 3′-/Cy5/rCrArA rCrCrU rCrGrA rC**rC**rA rCrCrG rCrArU rCrCrG	*KRAS wt*
DR7	–	5′-/ATTO532/GTT GGA GCT G**A**T GGC GTA GGC 3′-/ATTO647/rCrArA rCrCrU rCrGrA rC**rU**rA rCrCrG rCrArU rCrCrG	*KRAS G12D*
DR8	dArC	5′-/ATTO532/GTT GGA GCT G**A**T GGC GTA GGC 3′-/ATTO647/rCrArA rCrCrU rCrGrA rC**rC**rA rCrCrG rCrArU rCrCrG	*KRAS wt*
*Control duplexes series 1: DNA:RNA and DNA* + *LNA:RNA*			
DR9	–	5′-/Cy3/CTC CTG GG**C** TCA AGC AAT TCT 3′-/Cy5/rGrArG rGrArC rCrCr**G** rArGrU rUrCrG rUrUrA rArGrA	*EGFR np*
DR10	dCrU	5′-/Cy3/CTC CTG GG**C** TCA AGC AAT TCT 3′-/Cy5/rGrArG rGrArC rCrC**rU** rArGrU rUrCrG rUrUrA rArGrA	*EGFR wt*
DR11	–	5′-/Cy3/CAG CCT CCC A**C**G TAG CTG GGA 3′-/Cy5/rGrUrC rGrGrA rGrGrG rU**rG**rC rArUrC rGrArC rCrCrU	*EGFR np*
DR12	dCrC	5′-/Cy3/CAG CCT CCC A**C**G TAG CTG GGA 3′-/Cy5/rGrUrC rGrGrA rGrGrG rU**rC**rC rArUrC rGrArC rCrCrU	*EGFR wt*
DR13,DR13L	–	5′-/Cy3/ AGT AGA GAC + G + **C** + G GTT TCA CCA 3′-/Cy5/ rUrCrA rUrCrU rCrUrG rC**rG**rC rCrArA rArGrU rGrGrU	*BRAF np*
DR14 DR14L	dCrC +CdC	5′-/Cy3/AGT AGA GAC + G + C + G GTT TCA CCA 3′-/Cy5/rUrCrA rUrCrU rCrUrG rC**rC**rC rCrArA rArGrU rGrGrU	*BRAF wt*
DR15,DR15L	–	5′-/Cy3/GTT AGG TTG + G + T + C TCA AAC TCC 3′-/Cy5/rCrArA rUrCrC rArArC rC**rA**rG rArGrU rUrUrG rArGrG	*KRAS np*
DR16 DR16L	dTrC +TrC	5′-/Cy3/GTT AGG TTG + G + **T** + C TCA AAC TCC 3′-/Cy5/rCrArA rUrCrC rArArC rC**rC**rG rArGrU rUrUrG rArGrG	*KRAS wt*
*Control duplexes series 2: DNA:DNA and DNA* + *LNA:DNA*			
DD17-CTRL, DD17L1-CTRL	–	5′-GAG CGG AT + G + G + CG TAG GCA 3′-CTC GCC TAC CGC ATC CGT	*BRAF wt*
DD17L2-CTRL	–	5′-GAG CGG AT + G GCG TAG GCA 3′-CTC GCC TAC CGC ATC CGT	*BRAF wt*
DD17L3-CTRL	–	5′-GAG CGG ATG + GCG TAG GCA 3′-CTC GCC TAC CGC ATC CGT	*BRAF wt*
DD17L4-CTRL	–	5′-GAG CGG ATG G + CG TAG GCA 3′-CTC GCC TAC CGC ATC CGT	*BRAF wt*
DD17L5-CTRL	–	5′-GAG CGG AT + G + GCG TAG GCA 3′-CTC GCC TAC CGC ATC CGT	*BRAF wt*
DD17L6-CTRL	–	5′-GA + G CGG AT + G GCG TAG GCA 3′-CTC GCC TAC CGC ATC CGT	*BRAF wt*
DD17L7-CTRL	–	5′-GA + G CGG ATG GCG TAG + GCA 3′-CTC GCC TAC CGC ATC CGT	*BRAF wt*

*wt* wild type, *np* neutral polymorphism, *CTRL* control.

^a^Nucleotide pairs in the positions of single nucleotide polymorphisms are shown in bold; mismatched nucleotides in mutated oncogene models are underlined; duplexes with LNA nucleotides are indicated with L letter in the duplex name. Within the sequences, LNAs are indicated with a plus in front of corresponding nucleotide letter.

Each probe in DNA:RNA duplexes DR1–DR8 was labelled with the donor FRET at the 5′ end and acceptor FRET at the adjacent 3′ end of the complementary and mismatched RNA (Fig. 1c, Table 1). C12 linkers were used for attachment of fluorophores (C6 for amine-oligonucleotide and C6 for fluorophore reagent to be coupled, shown in Fig. 1c). The duplexes and their LNA variants were also tested without fluorophores. These sequences received a suffix “CTRL” in their sequence ID (Table 1, control duplexes series 2). The data obtained are shown in the Supplementary Table 5.

For mesoscopic modelling, additional DNA:RNA (abbreviated DR) and DNA:DNA (abbreviated DD) duplexes were designed (Table 1, control series 1 and 2; Supplementary Note 1). The designs have been done using public sequencing data (NCBI) and our previously published procedure^26,27^. DR9–DR16 were derived from the same oncogenes as the main duplex series DR1–DR8, but the position of the probes was moved upstream of the genes. Moreover, these duplexes contained different mutations (neutral polymorphisms, np) than the SNPs in DR1–DR8.

Effect of LNA was tested in several DR and DD duplex designs, mostly in those representing the *KRAS* oncogene due to the issues with its targeting DR5L, DR6L, DR15L, and DR16L and also *BRAF* duplexes DR13L and DR14L.

DD probes were shorter (18 nt) compared to the 21 nt long DRs. LNAs (1–3 modified nucleotide per duplex) were positioned in different positions along with the DD duplexes to account for the effect of the number and the sequence location of LNA on the stability and binding specificity. DD systems were all a full match; additionally, DD systems were labelled with individual fluorophores: Cy3, Cy5, ATTO532, and ATTO647. The resulting probes were investigated within the duplexes with complementary adjacent DNA and 5–10 nt long terminal DNA overhangs (Supplementary Table 5).

### FRET study of DNA:RNA duplexes

We started the study by FRET measurements of pre-annealed DNA/RNA tests systems DR1–DR8, DR5L, and DR6L (Table 1). The duplexes were hybridized and incubated with additional reagents at the desired concentration and ratio for 1.5 h (more details in the “Methods” section). Single-stranded oligonucleotides labelled with acceptor FRET were analysed, along with the duplexes, to account for FRET. The samples were analysed using a Roche Light Cycler 480 II multi-plate reader in a 364 well format. To observe FRET, the duplexes were excited with the donor’s excitation wavelength, and the fluorescence intensity of the acceptor was recorded. All samples were run in a duplicate with a coefficient of variance (CV) in fluorescence read out of 3.2–6.7% (Supplementary Table 2). The FRET efficiency was calculated using the following Eq. (1):^28^

1 $$E = \varepsilon _{\mathrm{{A}}}/\varepsilon _{\mathrm{{D}}} \times \left( {I_{{\mathrm{{AD}}}}/I_{\mathrm{{A}}} - 1} \right),$$

where *ε*_A_ and *ε*_D_ are the molar extinction coefficients of the acceptor and donor at the excitation wavelength, and *I*_AD_ and *I*_A_ are the fluorescence intensities at the acceptor emission wavelength in the presence and absence of the donor, respectively. When analyzing the data, we assumed that the changes in the FRET efficiency of duplex and duplex with additional reagents can be used to extract the information on the corresponding changes in oligonucleotide conformations^24^.

**Fig. 2:**
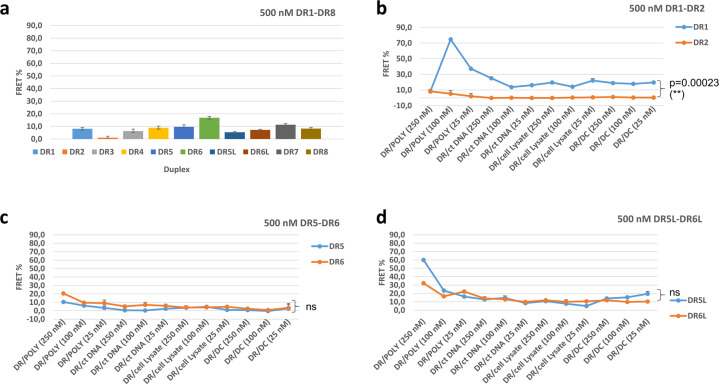
Comparison of calculated FRET efficiencies for duplexes DR1–DR8, DR5L, DR6L with and without different variables studied. **a** FRET efficiency for duplexes DR1–DR8 at a concentration of 500 nM, **b** FRET efficiency for duplexes DR1–DR2 at a concentration of 500 nM in presence of additives and with a variation in their concentration (250, 125, 25 nM), **c** FRET efficiency for duplexes DR5–DR6 at a concentration of 500 nM in presence of additives and with a variation in their concentration (250, 125, 25 nM), **d** FRET efficiency for duplexes DR5L–DR6L at a concentration of 500 nM in the presence of additives and with variation in their concentration (250, 125, 25 nM). Each measurement was performed twice (*n* = 2) and the error values were calculated. *P* values were calculated in R software using one-way ANOVA. Abbreviations: (**) indicates 99% confidence; (ns) = not statistically significant. DC = synthetic dsDNA crowder; POLY = poly-l-lysine; ct DNA = calf thymus DNA. *P* values are given for all data points in each series.

The resulting data on FRET efficiency (%) can be found in Fig. 2 and in the Supplementary Figs. 1–3. We expect higher FRET % for fully matched duplexes (DR1, DR3, DR5, DR7, and DR5L) vs. distorted mismatched duplexes (DR2, DR4, DR6, DR8, and DR6L)^24–26^. According to our results, without additional reagents FRET efficiency depends on the type of duplex, the presence of a mismatch in the target RNA, and the concentration (Fig. 2a). Mismatch specificity is high for the *BRAF* V600E probe (DR1 vs. DR2), while *KRAS* and *EGFR* show a smaller difference in FRET efficiency for matched (mutant) vs. mismatched (wild type) targets. Type of fluorophore had an effect on FRET efficiency as well. An ATTO FRET pair obtains higher FRET efficiency than a cyanine FRET pair. Mismatch discrimination by the ATTO *KRAS* duplexes DR7–DR8 is improved at duplex concentrations of 500 and 125 nM (Fig. 2a and Supplementary Fig. 2c, d) but not at the lowest concentration (25 nM, Supplementary Fig. 2e). Remarkably, adding three LNAs opposite to the SNP in the K*RAS* system does not lead to improved sensing of a mismatch by FRET. Oppositely, the LNA containing duplex with the mismatched target DR6L has a somewhat higher FRET efficiency than the fully matched analogue DR5L (Fig. 2a; data for DR5, DR6 vs. DR5L, DR6L).

Upon decreasing the concentration of the duplexes, FRET efficiency for 500 and 25 nM duplexes is lowered from 1–17% down to 0.6–3.1%, respectively (Supplementary Fig. 1). At the lowest concentration, the *BRAF* probe loses specificity toward the target, and matched and mismatched systems DR1 and DR2 have equal FRET efficiency of ~3% (Supplementary Fig. 1).

When analyzing the duplexes with additives, we observed a joined effect of the probe:crowder ratio and of the duplex concentration on FRET (Fig. 2b). At 500 nM D1, the duplex:poly-l-lysine ratio is crucial, with a 5:1 ratio giving a remarkably high FRET efficiency of 72%. With poly-l-lysine, mismatched system DR2 shows nearly no FRET at similar conditions, with the highest FRET being around 7.5% at a ratio of 2:1. The pattern changes when duplexes DR1 and DR2 are studied at 125 nM concentration. Here, we see lower FRET for DR1 than for the same duplex at 500 nM concentration. Now, the mismatched system DR2 with poly-l-lysine at ratios of 1:2, 1:1, and 5:1 (duplex:reagent) shows higher FRET efficiency than matched DR1 in similar conditions. At 25 nM DR1 and DR2, FRET efficiency is further reduced down to 0.1–4.2% with all added reagents. Overall, at 500 nM duplex and in the presence of additives, there is a statistically significant difference in FRET between matched and mismatched systems (Fig. 2b, *p* = 0.00023).

The system tested next was *EGFR* DNA/RNA DR3 and DR4, Table 1 and Supplementary Fig. 3. In this case, the mismatch dA → rC is located at the 3′-terminal region of RNA vs. a central location in *BRAF*. SNP can have a different location within a duplex according to the selected probe design. Terminally located SNP is a common design in enzymatic diagnostic methods, while better discrimination in hybridization assays occurs for SNP located in the middle of the strand^1,2^. Without additives, FRET efficiency is higher in the mismatched *EGFR* duplex DR4 than in matched DR3. Importantly, at 500 nM concentration of DR4, the addition of poly-l-lysine in the solution at a ratio of 20:1 (duplex to additive) increased the FRET efficiency of the system. Cell lysate and synthetic oligonucleotide in the ratio of 20:1 and 1:2 (duplex to additive) improved FRET efficiency of matched DR3 vs. DR4 at 125 nM duplex concentration. However, 100 nM poly-l-lysine increases the FRET efficiency of 125 nM duplex DR3 five-fold vs. DR4 (Supplementary Fig. 3b). This is contrary to a synthetic oligonucleotide and cell lysate additives, which at this duplex concentration increase FRET in both DR3 and DR4. At 25 nM duplex concentration, low FRET is observed in all systems. This is similar to the *BRAF* duplexes DR1–DR2 with nearly equal FRET efficiency in both DR3 and DR4 (Supplementary Fig. 3c). FRET efficiency by DR3 and DR4 in the presence of additives does not differ in statistical significance at all concentrations tested (Supplementary Fig. 3, *p* > 0.05).

Despite the similar principle for the probe design, *KRAS* G12D DNA/RNA duplexes DR5 and DR6 had a different behaviour than *BRAF* V600E DR1 and DR2 (Fig. 2a, c). At 500 nM concentration, the highest FRET efficiency was found at a duplex:poly-l-lysine ratio of 2:1. However, FRET efficiency is now higher for the mismatched system DR6 than DR5 (20% vs. 10%). Also, the FRET efficiency is approximately five-fold lower than for *BRAF* analogues (DR1 and DR2). The same is observed at 125 and 25 nM concentrations of DR5 and DR6 (Supplementary Fig. 1e, f). Opposite to DR3 and DR4, now another ratio of the duplex to poly-l-lysine has the highest FRET efficiency (5:1).

LNA is a bicyclic modified nucleic acid analogue that is often incorporated into synthetic oligonucleotide probes, and it improves their target binding affinity and specificity (Fig. 1a)^26^. To test the effect of LNA on the *KRAS* G12D system, we studied LNA-enriched DR5L and DR6L (Fig. 2d and Supplementary Fig. 2a, b). Overall, adding LNA changes both the pattern and the intensity of FRET in the *KRAS* G12D system in a concentration-dependent fashion. Using the duplex:poly-l-lysine at a 2:1 ratio, with 500 nM duplexes, leads to high FRET efficiency for matched DR5L (60%) and for mismatched DR6L (22%.) With other additives, FRET efficiency of DR5L and DR6L remains low and is somewhat similar in a different environment (<6%). At 125 nM, DR5L has a high FRET efficiency with an approximate 1:1 ratio with cell lysate (20%) and 18% with a 1:2 duplex:poly-l-lysine ratio; while at 25 nM system concentration and high (250 nM) poly-l-lysine, FRET is the highest in the series. In comparison to the mismatched analogue DR6L at 25 nM with high (250 nM) poly-l-lysine, FRET efficiency is lower (15% vs. 4%). For DR6L, 125 nM, FRET efficiency is highest at a 5:1 duplex:poly-l-lysine ratio (19%), and it is below 11% in all other conditions. Notably, neither unmodified nor LNA-enriched *KRAS* duplexes showed a statistically significant difference for match vs. mismatch systems (Fig. 2c, d; *p* > 0.05).

Further, we compared ATTO dyes to cyanines in the context of the *KRAS* G12D system (Supplementary Fig. 2c–e). The dyes were attached to the duplexes using similar C6-long linkers to avoid potential deviation in geometry (Fig. 1b, c). Quantum yield of cyanines and ATTO dyes attached to DNA probes are reported to be 0.04–0.27 and 0.8–0.9, respectively^29,30^. Their Förster radii are also comparable (ca. 57–63 Å). This has been considered when calculating FRET (formula (1)). FRET efficiency for ATTO systems DR7 and DR8 in all duplex concentrations is within the same range as the cyanine analogues DR5 and DR6. However, synthetic oligonucleotide and cell lysate now have a profound effect by increasing FRET efficiency in both matched and mismatched duplexes (Supplementary Fig. 2c–e).

In addition to the additives already tested, we decided to go a step further and to observe the effect of PEG on specifically chosen systems based on the already existing results. PEG is a commonly used crowding agent that is used to study synthetic oligonucleotide probes^31^. We tested DR1–DR2, DR5–DR6, and DR5L–DR6L in the presence of PEG2000 (Supplementary Fig. 4a). In the presence of PEG, FRET efficiency was lowered in all systems compared to other additives used in this study, and effective mismatch discrimination was observed only for DR1 vs. DR2.

Cancer cells over-actively divide^1,23^. Therefore, we hypothesized that nucleic acids themselves might act as a crowder. We accounted for this by studying specially chosen systems (DR1–DR2, DR5–DR6, and DR5L–DR6L) at 25 nM concentration and in the presence of cellular mRNA from a human leukaemia cell line (Supplementary Fig. 4b).

For the study, we estimated total intracellular mRNA concentration to be 2 nM upon extraction from single cell and reconstituted in 0.1 mL. This mimics an in vitro assay in the presence of total messenger mRNA form one cell. In practice, many more cells could be present in the sample, so the actual mRNA value could be higher, leading to an even more pronounced crowding effect. However, this high mRNA amount would interfere with our optical measurements; therefore, it was kept at 2 nM.

For DR1, FRET efficiency dramatically increased upon adding mRNA with a statistically significant difference (Supplementary Fig. 4b). At 2 nM mRNA concentration, our *BRAF*-specific probe was highly specific to the SNP V600E with FRET efficiency being 36% for DR1 + mRNA and 10% for DR2 + mRNA, respectively. *KRAS* probes DR5 and DR6 had the same FRET efficiency (10–11%); while with LNA, the FRET efficiency was higher in mismatched duplex DR6L (26%) vs. 21% in DR5L.

### UV Thermal denaturation and CD studies

Other segments that are very important for designing a system are the strength of binding and the duplex structure. To further examine those parameters, we conducted UV thermal denaturation and CD measurements for DR1 and its unlabelled DNA/RNA analogue (DR1-CTRL; Supplementary Information, Figs. 5–9). All UV thermal denaturation graphs showed an *S*-shaped monophasic transition, and the *T*_m_ values were in the range of 43–63 °C, being highest for the 500 nM duplex concentration (Supplementary Figs. 5–8, Supplementary Tables 3 and 4). The *T*_m_ data was then correlated with the distance between donor and acceptor FRET (Supplementary Fig. 4c, d). The distance *R*_D–A_ was calculated following the Förster equation as follows:

2 $${\mathrm{FRET}}\,{\mathrm{efficiency}}\,\left( {\mathrm{\% }} \right) = {\mathrm{100\% }} \times {\mathrm{1}}/\left( {{\mathrm{1}} + \left( {{R}_{{\mathrm{DA}}}/{R}_{\mathrm{0}}} \right)^{\mathrm{6}}} \right),$$

where *R*_0_ is a Förster radius (53 Å for Cy3/Cy5 pair)^32^.

We observed a linear correlation between the UV data and fluorometry with *R*^2^ values of 0.87 and 0.89 for 1000 and 500 nM D1, respectively (Supplementary Fig. 4c, d).

Following the CD results, there was no significant change in the structure between the control duplex and duplexes labelled with Cy3/Cy5 (Supplementary Fig. 9). Both systems showed a positive peak at 275 nm and a negative peak at 245 nm, which is a typical CD profile of a standard B-type duplex^33^. This implies that the probes in our design had a stabilization effect without a change in the structure of the duplex. On the contrary, CD spectra with poly-l-lysine and PEG (Supplementary Fig. 9b, e) showed a negative band at 210 nm and a positive band at 270 nm. The negative band at 210 nm is characteristic of the b-sheet of poly-l-lysine/PEG^34^. The appearance of the positive band at 270 nm with no negative peak at 240–255 nm points to an A-type duplex structure^33^. This indicates that the addition of poly-l-lysine and PEG may compact the DNA/RNA and change its conformation towards the more compact A-form.

### Mesoscopic modelling

**Fig. 3:**
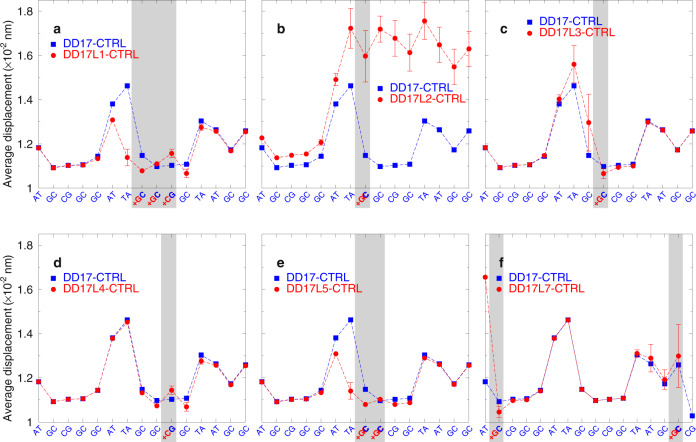
Representation of calculated average displacement profiles between base pairs. Panel **a** is for DD17L1, **b** for DD17L2, **c** for DD17L3, **d** for DD17L4, **e** DD17L5, and **f** DD17L7. Red bullets are for LNA-containing sequences, and blue boxes are for sequences with unmodified DNA. The location of the LNAs are indicated by the grey shaded area. The first and last base pairs, as well as the fluorophores, are omitted as their average displacement exceeds the vertical scale. The calculated uncertainties are shown as error bars only for the cases where they are larger than the symbol size. Sequences of test *t* bf di Tbl1.

**Fig. 4:**
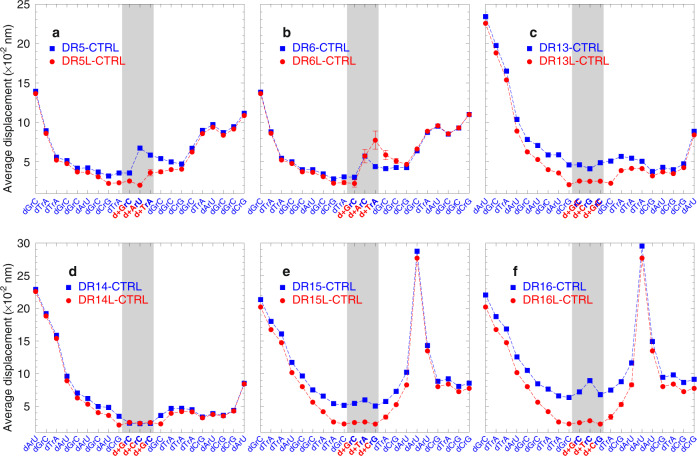
Calculated average displacement profiles between base pairs for DNA/LNA:RNA. Panel **a** is for DR5(L), **b** for DR6(L), **c** for DR13(L), **d** for DR14(L), **e** for DR15(L), and **f** for DR16(L). Red bullets are for LNA-containing sequences, and blue boxes are for sequence with unmodified DNA/RNA. The location of the LNAs are indicated by the grey shaded area. The calculated uncertainties are shown as error bars only for the cases where they are larger than the symbol size. For sequences of test systems, see Table 1 and Supplementary Table 15.

**Fig. 5:**
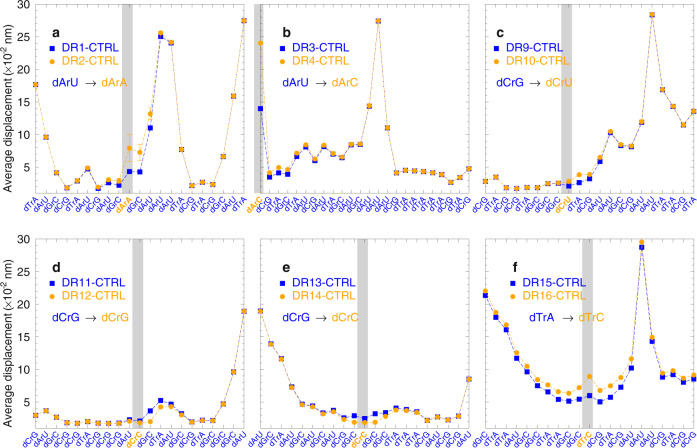
Calculated average displacement profiles between base pairs for DNA/LNA:RNA with mismatches. Panel **a** is for DR1(2), **b** for DR3(4), **c** for DR9(10), **d** for DR11(12), **e** for DR13(14), and **f** for DR15(16). Orange and blue bullets show the data for mismatched and matched sequences, respectively. The location of the mismatch substitutions are indicated by the grey shaded areas. The calculated uncertainties are shown as error bars only for the cases where they are larger than the symbol size. For sequences of test systems, see Table 1 and Supplementary Table 15.

Next, we carried out mesoscopic calculations on the model duplexes^35^. This approach processes the measured melting temperatures to extract information about the hydrogen bonding, via a Morse potential, and stacking interactions. The details on the model duplexes and on the computing procedure are given in the “Methods” section and Supplementary Information. The sequences of the control systems can be found on the horizontal axis of Figs. 3–5 and in the Supplementary Tables 5–7 and 15. To initially establish the hybridization parameters, we used the data for our test system that was formed by oncogene-specific oligonucleotide probes and DNA targets with and without terminal overhangs.

In brief, we started with optimizing the duplex parameters without fluorophores, using as input values the previously calculated parameters at a salt concentration of 69 mM^36^. The calibrated parameters for DD series (Table 1) were used in the next step where the fluorophores were introduced. Finally, we included LNA (indicated with a plus sign in front of the corresponding nucleotide letter) into the *KRAS* system and performed a subsequent optimization round using average Morse potentials of the 20 best hits from the previous computing step. The results are presented in Fig. 3, where we show the average displacement between base pairs calculated with the new parameters^37,38^. These types of displacement profiles provide a qualitative visualization to help understand the interplay of the on-site and nearest-neighbour parameters. Despite finding a substantially increased Morse potential depth for +GC, beneficial stability was observed only in few specific cases. In particular, +GC followed by +G (+G+G) provided a moderate increase in stabilization. However, in one specific situation, shown in Fig. 3b, a +GC followed by GC (+GG) was found to be unstable due to very low stacking parameters. This instability in DD17L2-CTRL results in large average displacements, shown in Fig. 3b, which strongly affect the 3′ side of this sequence. This means that a generic design approach, placing the central part of the probe opposite to the mutation, is not working well in the case of sequences rich in GC pairs. In particular, simply adding LNA does not always improve the situation and may lead to unforeseen instabilities.

Next, we performed a similar mesoscopic analysis of the measured melting temperatures of the DNA/LNA:RNA sequences. Here, we started with low-salt DNA/RNA parameters obtained from an independent set of temperatures in a similar way to how we had previously calculated for high-salt sequences^39–41^. Mismatches and canonical base pairs were treated in exactly the same way, the only differences were in the resulting parameters after the optimization procedure. The new parameter set indicates that especially the dArU base pair, that was previously found to have the lowest Morse potential depth of 28 meV for high-salt concentrations, becomes even weaker to a mere 12 meV. Figure 4e and f show the calculated sequence displacement profiles, and these clearly show the effect of the weak dArU base pairs with pronounced openings. Different from the DNA:DNA systems shown in Fig. 3, the DNA:RNA sequences stabilize in the presence of LNA modifiers, except for d+ArC and d+TrA, as shown in Fig. 4b.

In Fig. 5, we show the displacement profiles in the presence of mismatched DNA/RNA. It is known that DNA/RNA with a high deoxy-pyrimidine content (dC and dT) are much more stable^39^. However, it is unclear if the same reasoning can be applied to mismatches in the present buffer conditions. From experiments in high sodium content, it is known that dArA is unstable^42^ and this is also what we observe in Fig. 5a when dArU, which is ordinarily the least stable canonical base pair, is replaced with dArA. On the other hand, the dCrC, which in principle should be even less stable^42^ than dArA, turns out to be equivalent to dCrG, as shown in Fig. 5e. Similarly, dTrC which also is known to be one of the least stable DNA/RNA mismatches^43^ also shows an important destabilization in Fig. 5f. One important aspect that affects the stabilization is the presence of multiple dArU, which shows spikes of pronounced helix openings in Fig. 5a–d. Here, it becomes clear that for probe design these regions should be avoided whenever possible. Note that the DNA/RNA modelling was performed with a lower amount of melting temperature data than for DNA/DNA; therefore the resulting parameters have larger uncertainties. Nevertheless, the increased uncertainty appears to have little effect on the average displacements of Figs. 4 and 5, as only few positions had larger error bars.

### Bead-bait RNA genotyping assay

**Fig. 6:**
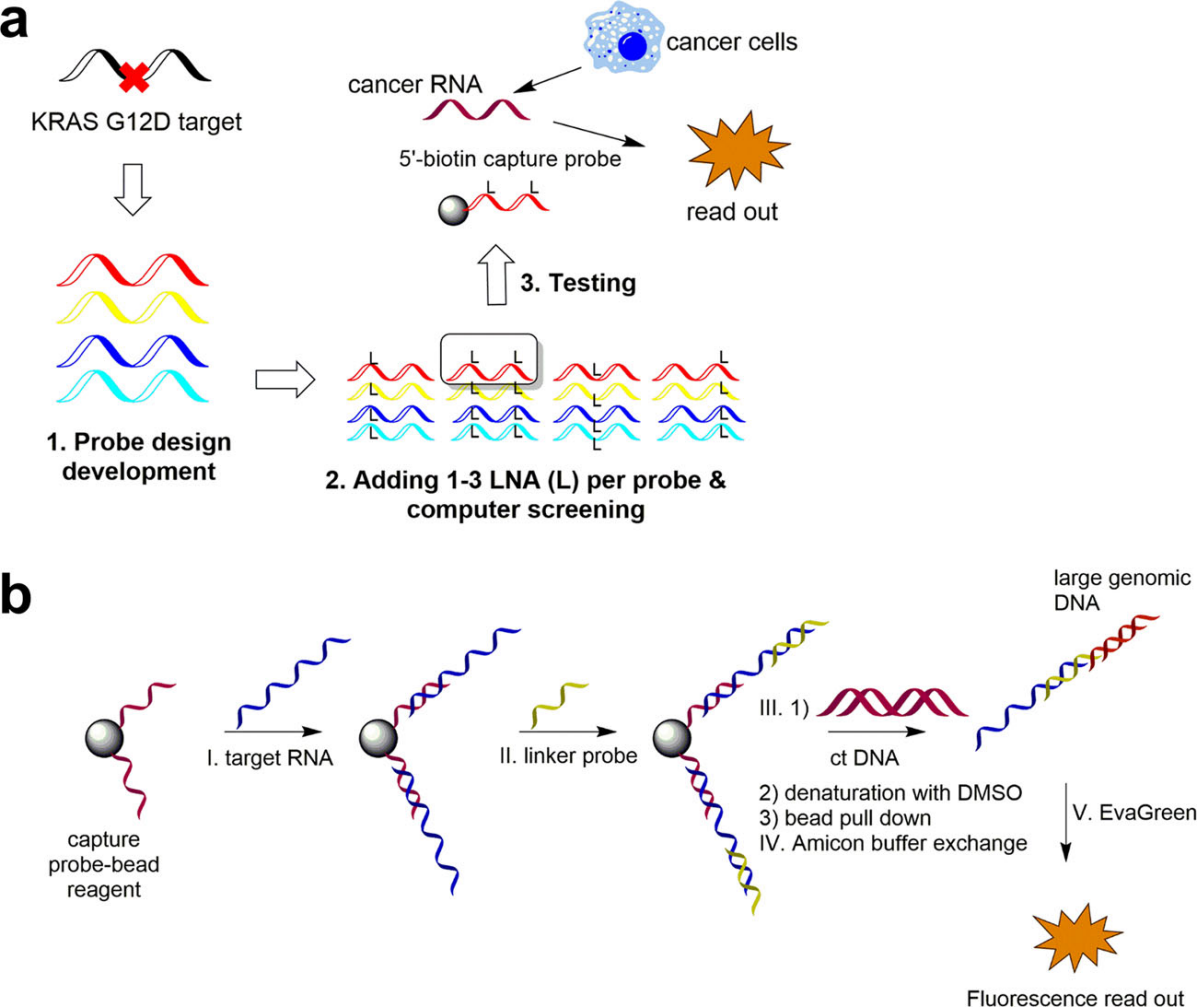
Bead-bait hybridization assay for detection of *KRAS* G12D mutation. **a** An approach to developing genotyping probes for *KRAS* G12D, **b** Main steps of the assay including (I) formation of bead-capturing probe followed by catching or “purification” of the target RNA by washing the non-binding genetic material; (II) hybridization with specific linker probe to link target RNA with ct DNA followed by washing step; (III) ct DNA addition and linkage to the linker probe followed by a washing step then DMSO denaturation and separation from the bead-capturing probe (IV) re-establishment of the hybridization conditions with buffer exchange and fluorescence readout using intercalating Eva Green dye. LNA (L) locked nucleic acid.

In this step, we applied the developed oligonucleotide hybridization model to the design of target-specific genotyping probes. We selected a bead-bait RNA detection as a robust strategy to test our new approach to probe design. The workflow for probe preparation and the scheme of the assay are shown in Fig. 6. Our main goal was to detect RNA from *KRAS* G12D oncogene, which is known to be a very challenging target for PCR. Nevertheless, to establish the assay, we initially tested our approach on two commonly detected mutations: *BRAF* V600E and *EGFR* L858R. Our assay was benchmarked to LNA RT-qPCR^44–46^ and to next-generation sequencing (see “Methods” section and Supplementary Note 3 for details).

For *BRAF* and *EGFR*, already existing designs were used (shown in Table 1; DR1 and DR3, respectively). For the *KRAS* probes, we developed seven main designs where we varied the position of the mismatch (G12D mutant → wild-type replacement) along the strand (Supplementary Table 19). The designs were based on the available data for PCR and sequencing primers where it was shown that the additional mismatches and a terminal position of a target mismatch are beneficial for the discrimination of wild-type vs. mutant amplicon^44–46^.

To improve the initial design, we considered adding LNA^45^. However, according to our data in the FRET study of DNA:RNA duplexes, incorporating LNA has to be done in certain positions to avoid negative effects on mismatch discrimination. To approach this problem, we developed a theoretical dataset where we included LNA in all possible positions along with the probes in a number from one to three LNA per probe (Supplementary Table 20). The obtained probes were screened for RNA binding using our developed binding potentials described in the “Mesoscopic modelling” section, SI. According to this screening, the original probe design with a central location of the *KRAS* G12D mutation had the poorest discrimination for mutant vs. wild-type RNA, whereas adding a mismatch and shifting its position to the terminal area of the probe improved the discrimination (Supplementary Table 20).

To validate our prediction, we selected 10 probes with the most and least promising mismatch discrimination values according to the theoretical model and tested them against synthetic *KRAS* RNA oligonucleotides. We observed good agreement of the experimental *T*_m_ values with the theoretical data (deviation below 1.5%, see Supplementary Table 21). When the crowders DC and RNA were added, the discrimination decreased only slightly. This would be of importance for the assay, given that the entire cellular RNA would be added as a sample at the initial step and ct DNA upon installing the signal boosting DNA and the linker probe (Fig. 6b).

Inspired by this result, we proceeded with the bead-bait assay and benchmarked it to RT-qPCR that uses commercial LNA-enriched primers. For the study, we purchased commercial cancer cell lines. The mutation status in these cells has been tested by next-generation sequencing. Next, selected capture probes and controls for the bead-bait assay were obtained as 5′-biotinylated LNA/DNA sequences. At the first step, a biotinylated capture probe was applied to streptavidin-coated magnetic beads. Next, RNA samples from cancer cells (HT-29) or negative controls (MRC-5) were incubated with the capture-bead (“bait”) reagent. The beads were washed with 1X PBS several times, to eliminate off-target binding, and subjected to binding with a linker probe. The linker probe was designed to bind a part of a cancer RNA (12–16 nt region) that had a spacer (T4) and a complementary oligonucleotide to the large genomic ct DNA (over 100,000 nucleotides long dsDNA). After the cold denaturation with 7% DMSO^47^ and rapid buffer exchange with an Amicon device to remove the DMSO, we added a fluorophore to the sample. We selected Eva Green dye as a convenient fluorophore that discriminates the dsDNA vs. ssDNA with over a 100-fold light up of fluorescence (excitation/emission wavelength of 490/510 nm). Our previous studies showed that one molecule of Eva Green dye binds to a five-nucleotide long dsDNA fragment^25^. Thus, the presence of ct DNA in a sample leads to a bright fluorescence response. Using a calibration curve for a known amount of ct DNA signal “booster” (Supplementary Fig. 10), we could then estimate the concentration of the RNA in the cancer cell samples.

Our data for *BRAF* and *EGFR* mutations are shown in Supplementary Table 22. In good agreement with LNA RT-qPCR and with sequencing data, *BRAF* V600E and *EGFR* L858R mutations were identified at 315 and 122 pM concentrations, respectively, with a deviation from the bead assay to RT-qPCR below 2%. This proved that our bead-based assay worked well for these two oncogenes.

We proceeded with the analysis of the *KRAS* G12D mutation. According to sequencing, HT-29 cells were *KRAS* G12D mutated, and MRC-5 cells were wild-type. With regard to probe design, we took the three best hits from the probe designs: DR23 (#208, 283), DR26 (#M208, 283), and DR29 (M17, M97). These probes discriminated the mutant vs. wild-type by over 5 °C difference in *T*_m_. Besides these probes, we had control captures (DR5 and DR21 variants) that were less effective according to our prediction (Δ*T*_m_ 0–2 °C).

As can be seen in Supplementary Table 23, we had over a four-fold higher signal for probes DR23, DR26, and DR29, vs. RT-qPCR (104–120 pM ± 1.8–3.6% vs. 20 pM ± 14.1%) in cancer-positive HT-29 cells. However, in MRC-5 RNA, probes DR23, DR26, and DR29 showed no signal (<10 pM ± 5.4–11.3%) vs. the 23.33 pM ± 11.25% result for RT-qPCR. Using control capture probes DR5, DR19, and DR21, we had an increased signal for HT-29 in the bead assay, up to 163 pM ± 1.6%, most likely because a wild type RNA had been bound. Nevertheless, control probes did not give an increased signal in the negative control MRC5 (<10 pM ± 6–17%).

When diluting the mutant RNA with a wild type, the bead assay with the selected probes DR23, DR26, and DR29 allowed detecting as little as 2.4 pM ± 3.1% of the mutant (Supplementary Tables 24–26). LNA RT-qPCR assay resulted in 2–3-fold lower sensitivity than our probes DR23, DR26, and DR29, with 12.5 pM ± 2.3% mutant RNA that could be detected (Supplementary Table 27). In bead-based assays, target dilution results followed a linear trend above 3.125% of the mutated target. For LNA RT-qPCR, linear trend was observed across all concentrations of the mutated target (Supplementary Fig. 11).

## Discussion

The accurate prediction of target recognition by synthetic oligonucleotide probes is in high demand to secure efficient clinical diagnostics and research of pathogens, inherited diseases, and cancer. In this work, we approached three human oncogenes, *BRAF*, *KRAS,* and *EGFR*, and studied the mutation-specific probes as duplexes with a fragment of cancer RNA. Based on the plethora of reports by us and others, duplexes with RNA have a higher *T*_m_ than corresponding DNA:DNA duplexes. The studies also show that DNA:RNA duplexes are potent displacement systems for a wide range of assays^1,2^.

Our work addresses an unmet need in the nuanced study of hybridization properties of synthetic oligonucleotide probes towards RNA targets^48–51^. Based on our results, we conclude that careful consideration should be put into the selection of a suitable crowder/additive. Cell lysate shows little to no effect in most systems that we have tested. Poly-l-lysine and PEG2000 have different effects depending on the duplex sequence and concentration. The addition of nucleic acids has not been studied before, and for the first time, we report that synthetic oligonucleotides themselves can act as crowding agents in a sequence-dependent and concentration-dependent fashion.

In our study, we chose FRET as a major read out for studying the effect of crowders on DNA:RNA systems. Given that fluorophores are attached to oligonucleotides via C12 linkers, we estimate the distance between them for a fully matched duplex to be 36 Å, eliminating the risk for contact quenching.

Having replaced the cyanine FRET pair with ATTO, we confirm that the dyes also have an effect on target recognition by the probes. Herein, cyanine probes had better mismatch sensitivity according to FRET measurements. ATTO dyes have high FRET efficiency in mismatched *KRAS*. This might be accounted for by the specific sequence of *KRAS* leading to the aforementioned challenges with its detection.

FRET efficiency is affected by decreased duplex concentration and by additives. This could be caused by changes in the distance between fluorophores and in their environment. The distance to fluorophores correlates with the duplex structure, which makes us hypothesize that the dilution and certain additives decrease the amount of fully hybridized species in the sample^52^.

Comparing FRET analysis with conventional UV thermal denaturation data, we see that there is a good match in FRET-based distance between the fluorophores and the *T*_m_ values. This finding confirms the fact that the FRET study is reliable and can be used to assess oligonucleotide hybridization below the limit of target detection for the less sensitive UV method.

Among human oncogenes, *KRAS* is known to be a very challenging task for genotyping, and our study confirms this^4^. The most commonly used methods of detection for cancer mutations, PCR and sequencing, have suboptimal sensitivity in case of *KRAS* diagnostics^44–46^. This becomes especially critical when the sample contains a small fraction of malignant cells. Alternative methods with higher sensitivity have been proposed in the literature^53^. For instance, Arcila et al. report higher analytical sensitivity of LNA-modified PCR, followed by sequencing and mass spectrometry analysis of *KRAS* compared with standard sequencing. Using the LNA-PCR sequencing, they managed to detect 6% more additional *KRAS* mutations in colorectal carcinoma associated with primary resistance to EGFR inhibitors^44^. In addition, a study by Ishige et al. states that the use of an LNA probe as a wild-type blocker in Sanger sequencing increases the sensitivity to detect *KRAS* mutations. While the conventional Sanger sequencing successfully detected mutations with 10–30% frequencies, the LNA-Sanger sequencing showed efficient sensitivity in the detection of *KRAS* mutations with 5% frequency^54^. Nevertheless, all of the previously reported LNA-based techniques require enzymatic target amplification^45,46,53,54^, and this is what differentiates them from our method. Importantly, amplification-free methods provide us with absolute amounts of mutated targets rather than the qualitative/semi-quantitative results achieved by PCR and sequencing.

In our amplification-free study system, adding LNA modifications does not help the poor mismatch discrimination in the *KRAS* G12D system by fluorescence. The computational analysis brings further light on this issue, showing that *KRAS* RNA is poorly discriminated by the probe due to a CG region internally within the probe:target complex that leads to moderate to no effect of LNA on mismatch. Notably, LNA-enriched PCR probes perform poorly in detecting *KRAS* mutations compared to the amplification-free assay that uses rationally designed LNA/DNA reagents as well. The main issues with LNA RT-qPCR were disagreement with sequencing results and, in particular, a false positive signal in MRC-5 healthy cells. This demonstrates that the rational approach to probe design that we describe not only allows us to detect a higher portion of the mutated cancer RNA, but it is also applicable in a simple assay that also provides us with absolute target amounts in <2 h.

In conclusion, we successfully applied a series of synthetic oligonucleotide probes, labelled with FRET pairs, as tools to study nucleic acid hybridization of human oncogenes. We supported our fluorometry data with UV thermal denaturation and CD analyses and by calculating the model Morse potentials and stacking properties. A novel aspect of our work is using human oncogenes of different sequences in systematically alternated conditions that mimic an intracellular environment and in vitro samples upon purification of DNA/RNA from cells. We show that *BRAF* V600E is the most robust oncogene that can be well discriminated by fluorescently labelled oligonucleotide probes. In turn, *EGFR* and especially *KRAS* pose the ultimate challenges to probe design, due to low stacking parameters of oncogene:probe complexes.

Our work points out another important issue of nucleic acid-based assays, namely the high influence of the chemical nature and the amount of additive/crowder agents on the optical properties of the system. In particular, the commonly used PEG2000 and cell lysate gave very different results which were further complicated by sequence and concentration effects. Notably, nucleic acids themselves (genomic DNA, mRNA, and synthetic dsDNA) act as crowders with a profound stabilizing effect at low concentrations.

This study can be translated into practical applications in multiple ways. First, a new framework for extended assessment of hybridization properties can be used to develop specific oligonucleotide probes. Next, the reported DNA:RNA duplexes can act as displacement probes for in vitro and cellular assays^1–4^. Third, and most importantly, this work can raise awareness and guide the selection of study conditions for oncology-related sensors^5,6^.

## Methods

### General

Reagents and solvents obtained from commercial suppliers were used as received. All nucleic acid compounds were obtained from Integrated DNA Technologies, Inc., Iowa, USA, and Qiagen, Germany. Phosphate-buffered saline (product number P4417-50TAB) used for annealing, Calf thymus DNA (ct DNA) (cat no. D1501), poly-l-lysine, and PEG were ordered from Sigma. Leukaemia (K-562) total mRNA was purchased from Thermo Fisher Scientific.

IE HPLC was performed using the Merck Hitachi LaChrom instrument equipped with a Dionex DNAPac Pa-100 column (250 mm × 4 mm). Elution was performed starting with an isocratic hold of A-buffer and C-buffer for 2 min followed by a linear gradient to 60% B-buffer over 28 min at a flow rate of 1.0 mL/min (A-buffer, MQ water; B-buffer, 1 M NaClO_4_; C-buffer, 25 mM Tris–Cl, pH 8.0) (see Supplementary Table 1)

Streptavidin-coated magnetic beads were obtained from NEB (cat. no. S1420).

The cells were purchased from ATCC (MRC-5 (ATCC^®^ CCL-171); HT-29 (ATCC^®^ HTB-38)) and grown in a media for 2 weeks with splitting every 4th day until a stable cell line was obtained.

Cell processing, RNA isolation, NGS, and RT-qPCR were conducted using Qiagen kits (Qiagen RNeasy Cat No. 74104; RT2 HT First Strand Kit 330404 and UCP HiFidelity PCR Kit 202742) and Illumina library preparation (TruSeq 20020595) following the manufacturer’s standard protocols. The sequencing was carried out using Illumina 2000 equipment. The data handling was done using fully automated genome annotation for human oncogenes by Cloud Computing, San Francisco, USA.

UV Thermal denaturation measurements were performed on a DU^®^800 spectrophotometer, Beckman Coulter. CD spectra were recorded on a JASCO-815 CD spectrometer equipped with a CDF 4265/15 temperature controller. Fluorescence measurements were carried out on a Roche Light Cycler 480 Real-Time PCR Machine in a 364 well plate format.

### Design and annealing of duplexes

Probes were designed (see Supplementary Data 1) using public sequencing data via NCBI:*BRAF* (NM_001354609.1); *KRAS* (NM_033360.3), and *EGFR* (NG_007726.3). The uniqueness of the designed probes to the targets has been confirmed using Stanford University Sequence Uniqueness Software. The dsDNA used as an additive reagent was as follows: 5′-d(TGT GGT AGT TGA GCG GAT GGC GTA GGC A)-3′: 5′-d(TGC CTA CGC CAT CCG CTC AAC TAC CAC A)-3′. For annealing, two oligonucleotide strands were mixed in an equal molar ratio in 1X PBS, pH 7.2, vortexed, kept at 85 °C for 10 min followed by cooling to room temperature over 4 h.

Pre-annealed duplexes were mixed with an additive at desired concentrations and incubated at 37 °C for 1.5 h followed by immediate measurement of UV *T*_m_, CD and/or fluorescence.

UV Thermal denaturation measurements were performed on a DU^®^800 spectrophotometer, Beckman Coulter, with a ramp temperature increase of +0.1 °C/min. CD spectra were recorded on a JASCO-815 CD spectrometer equipped with a CDF 4265/15 temperature controller. Fluorescence measurements were carried out on a Roche Light Cycler 480 Real-Time PCR Machine in a 364-well plate format.

### UV Thermal denaturation studies

DR1 and DR1-CTRL were analysed at seven different concentrations (500, 400, 250, 125, 50, 25, and 5 nM). Reported *T*_m_ values present the maximum of the first derivative of the curve and are an average of two measurements with deviation ±0.5 °C.

### Mesoscopic modelling

Mesoscopic modelling was used to extract oligonucleotide parameters from the measured duplex melting temperatures. The procedures followed are outlined elsewhere^35,36^ and summarized in Supplementary Note 1. For the DNA:LNA/DNA sequences, the data set, shown in Supplementary Table 5, was separated into sequences considering their specific characteristics: overhangs, fluorophores, and LNA modifications. The overhangs and fluorophores were handled as if they were additional bases following a procedure we had developed previously for cyanine markers^7^. Considering each of these properties, the associated parameters were calculated in four separate steps that are outlined in detail in Supplementary Note 1. We started with optimizing the duplex parameters without fluorophores, using as input values the previously calculated parameters at a salt concentration of 69 mM^36^. The calibrated parameters for DD series (Supplementary Tables 8 and 9) were used in the next step, where the fluorophores were introduced (Supplementary Tables 10–12). Finally, we included LNA (indicated with a plus sign in front of the corresponding nucleotide letter) into the *KRAS* system and performed a subsequent optimization round using average Morse potentials of the 20 best hits from the previous computing step, shown in Supplementary Tables 13 and 14. The resulting parameters for the DNA:LNA/DNA sequences are shown in Supplementary Tables 8–14. The procedure for the DNA:LNA/RNA was simpler due to the lack of overhangs, and it is detailed in Supplementary Note 2. For the canonical DNA/RNA parameters we used another data set at low salt concentration^41^, which in turn was used as a base set for calculating the Morse and stacking parameters with fluorophores, LNA, and mismatches, shown in Supplementary Tables 16–18. The displacement profiles shown in Figs. 3–5 were calculated, as detailed previously^37^, based on a method developed originally by Zhang et al. ^38^.

### Bead-bait hybridization assay

At each assay step, the beads were mixed by vortexing for 10 s every 2 min to avoid sedimenting. A biotinylated capture probe (0.2 nmol) was added to streptavidin-coated magnetic beads (50 µL) in 1X PBS. After 10 min, the supernatant was removed, and the beads were washed twice with 1xPBS (100 µL). An RNA sample (20 ng/µL; 5 µL) was added, and the mixture was kept at room temperature for 20 min, followed by removing the supernatant and washing the beads (2× 1X PBS, 100 µL). The linker probe (0.2 nmol) was added for 20 min at room temperature, followed by three washes at elevated temperature (100 µL for each wash with buffer pre-heated to 42 °C) and adding ct DNA (2 mg/mL, 5 µL) for 20 min at room temperature.

Linker probe sequences were designed to be located 20–50 nucleotides upstream to mutation-specific capture probes, as described^55^, and they were as follows:

*BRAF*, 5′-d(A+TC AG+T TTG AAC A+GT+TG TTTT **GA**+**T GG**+**G AAT A**+**CC AGA C**+**CA C**+**CTG**)

*KRAS*, 5′-d(A+CG A+AT ATG ATC+CAA C+AA TTTT **GA**+**T GG**+**G AAT A**+**CC AGA C**+**CA C**+**CTG**)

*EGFR*, 5′-d(A+TA+TAT AAT GTG A+CT T+CA TTTT **GA**+**T GG**+**G AAT A**+**CC AGA C**+**CA C**+**CTG**)

where LNAs are indicated with a plus in front of the corresponding nucleotide; the sequence part binding to ct DNA is shown in bold. Ct DNA genome accession code used for the linker sequence design was NC_037330.1, GI: 1378962611. The part of the linker probe shown in bold has a higher affinity to a ct DNA (+) strand than its complement. Therefore, a linker probe acts both as target RNA-binding reagent and as invader probe to ct DNA.

The supernatant was removed and after four washes (100 µL 1X PBS); then we added 7% DMSO in 1X PBS (50 µL), which denatured the duplex^47^. The supernatant was removed and recovered in 1X PBS using an Amicon 3 MWKO device (Millipore). The final volume of the recovered sample was 10 µL; 0.6 µL Eva Green (Biotium, 20X stock) fluorophore was added and the fluorescence was measured immediately using a 384-well plate Roche Light Cycler 480 plate reader. The data was analysed using Light Cycler fluorescence analysis software following the manufacturer’s recommendations.

Reverse transcription (RT) and LNA/DNA PCR primers were synthesized by Qiagen following the previously reported considerations^45,46^. Per PCR reaction, we used 100 ng RNA extracted from cell lines with an average length of 1.1 kb, as determined by a Qiasymphony SP instrument.

Reverse transcription was carried out as follows: mix and incubate at 42 °C for 1 h the following components: RNA target (5 µL; 20 ng/µL), the corresponding RT primer (50 µM; 2 µL); RT supermix (Qiagen) (10 µL); and 3 µL RNAse-free water (20 µL total volume). The enzyme was inactivated at 85 °C for 5 min. Each experiment has been carried out in triplicate.

Reverse transcription gene-specific primer sequences were as follows:

*BRAF*, 5′-d(AGA GCT CTT ATC AAT TTG TTG CAA CGA AC)

*EGFR*, 5′-d(AGA GCT AGT ATA GAG GTC TTA CAC ATT TTT GT)

*KRAS*, 5′-d(AGA GCT ACT TTA TAA GCC ATA GAC ACT ATA GT)

Upon RT completion, DNA was purified by an Amicon device, MWKO 100 kDa, and reconstituted into ultra-pure water to a total volume of 5 µL (Millipore/Sigma Z648043). PCR was carried out immediately using the following primer pairs:

LNA-DNA PCR primers:

BRAF, Forward 5′-d(GCC TGA AGA CCT CAC AGT AA); Reverse 5′-d(ACT CCA TCG AGA TTT C+T);

*KRAS G12D*, Forward 5′-d(GTG GTA GTT GGA GCT G+T); Reverse 5′-d(AGA GTG GCC CTT GAC GAT ACA).

*EGFR* L858R, Forward 5′-d(GCA TGT CAA GAT CAC AGA TT); Reverse 5′-d(CCA GAC CCA AGT TTG GCC C+T), LNA is marked with a plus in front of corresponding letter. Nucleotides opposite to the mutation in the target are underlined.

### LNA PCR

The real-time amplification was done on a LightCycler 480 II system (Roche Applied Science, Indianapolis, IN). Each qPCR reaction was performed in triplicate within a sealed LightCycler 480 Multiwell Plate 96 (Roche) in a 30 μl volume mixture containing template cDNA from RT step (5 μL; 50 ng), primer mix 10 µM (5 μL), PCR supermix (Qiagen), and miliQ water. The PCR amplification was carried out as follows: 10 min at 95 °C and 45 cycles of 30 s at 95 °C and 45 s at 62 °C. LightCycler 480 software release 1.5.0 was used for mutation identification by advanced relative quantification analysis. Sample crossing point (Ct)-values were converted to concentration using internal calibration provided with the Qiagen supermix kit.

### Statistical analyses

Data was checked for normality of distribution using the Shapiro–Wilk test in R software^56^. One-way ANOVA was applied to the data sets in R software^56^. The results with a *P*-value over 0.05 were considered significant^56^.

## Supplementary information

Supplementary Information

Description of Additional Supplementary Files

Supplementary Data

## Data Availability

The datasets generated during and/or analysed during the current study are available from the corresponding author on reasonable request.

## Code availability

The datasets generated during and/or analysed during the current study are available from the corresponding author on reasonable request. The software and mesoscopic modelling parameters are available at http://tinyurl.com/tfregufmg, see also ref. ^37^.
