# Supplementary material for: Optical and theoretical study of strand recognition by nucleic acid probes
Source: Commun Chem. 2020 Aug 11;3:111. doi: 10.1038/s42004-020-00362-5 (PMC9814704; doi:10.1038/s42004-020-00362-5)
Supplement: Supplementary file 1 — Supplementary Information [file 42004_2020_362_MOESM1_ESM.pdf]

Supplementary Information for the Paper:

Optical and theoretical study of strand recognition by nucleic acid probes

Ivana Domljanovic, Maria Taskova, Pamella Miranda de Moura, Gerald Weber and Kira Astakhova*

* E-mail: kiraas@kemi.dtu.dk; Tlf. +4593513552

Table of Contents

Table of Contents..1

Oligonucleotides..2

Fluorescence measurements..3

Tm studies.. 9

CD measurements 15

Supplementary notes 1-2. Parameter optimization 17

Supplementary note 3. Bead-bait hybridization assay 31

Oligonucleotides

All the strands were analyzed by IE HPLC and their purity exceeded 94% which has been considered sufficient for the studies.

Supplementary Table 1. Representative purity of oligonucleotides confirmed by IE HPLC.

Code	Sequence, 5'->3'	Retention time, min	Purity,%
DR1-CTRL	TAG CTA CAG AGA AAT CTC GAT	7.8	96
DR1-CTRL	rArUrC rGrArG rArUrU rUrCrU rCrUrG rUrArG rCrUrA	12.3	98
DR1,DR2	/**Cy3**/ TAG CTA CAG AGA AAT CTC GAT	16.2	95
DR1	rArUrC rGrArG rArUrU rUrCrU rCrUrG rUrArG rCrUrA /**Cy5**/	20.1	94
DR2	rArUrC rGrArG rArUrU rUrCrA rCrUrG rUrArG rCrUrA /**Cy5**/	20.4	94
DR5,DR6	/**Cy3**/ GTT GGA GCT GAT GGC GTA GGC	15.5	95
DR5	rGrCrC rUrArC rGrCrC rArUrC rArGrC rUrCrC rArArC /**Cy5**/	21.1	97
DR6	rGrCrC rUrAC rGrCrC rArCrC rArGrC rUrCrC rArArC/**Cy5**/	21.0	96
DR5L,DR6L	/**Cy3**/ GTT GGA GCT +G+A+T GGC GTA GGC	12.2	98
DR7,DR8	/**ATTO532**/ GTT GGA GCT GAT GGC GTA GGC	10.5	96
DR3,DR4	/**Cy3**/ ACT GTA CAT GAG AAA CTT TTT CTC	9.5	94
DR3	rGrArG rArArA rArArG rUrUrU rCrUrC rArUrG rUrArC rArGrU /**Cy5**/	22.4	96
DR4	rGrArG rArArA rArArG rUrUrU rCrUrC rArUrG rUrArC rArGrC /**Cy5**/	21.8	95
DC	TGT GGT AGT TGA GCG GAT GGC GTA GGC A	9.4	95
DC	TGC CTA CGC CAT CCG CTC AAC TAC CAC A	10.1	95

Fluorescence measurements

Assays have been done in duplicate for all probes in all tested conditions. Representative coefficient of variance (CV) values (%) for randomly selected 10 systems are given in Supplementary Table 2.

Supplementary Table 2. Representative results for duplicate fluorometry measurement and CV calculation.

System	Concentration	Additive, concentration	FRET % - Measurement 1; 2	CV,%
DR1	125 nM	None	18.1; 18.7	1,6
DR3	25 nM	None	0.6; 0.64	3,2
DR4	500 nM	None	4.7; 4.5	2,2
DR5	500 nM	Cell lysate, 25 nM	4.6; 4.4	2,2
DR5L	500 nM	None	6.8; 7.2	2,9
DR6	25 nM	POLY, 250 nM	2.1; 2,4	6,7
DR7	125 nM	ct DNA, 25 nM	5.3; 4.9	3,9

Supplementary Figure 1. Representation of FRET efficiency values for tested DNA/RNA duplexes DR1-DR6 with or without additional reagents. POLY = poly-L-lysine; ct DNA = calf thymus DNA; DC = synthetic dsDNA crowder. ns = not significant ($p>0.05$); (**) = statistically significant with $p < 0.01$.

A)

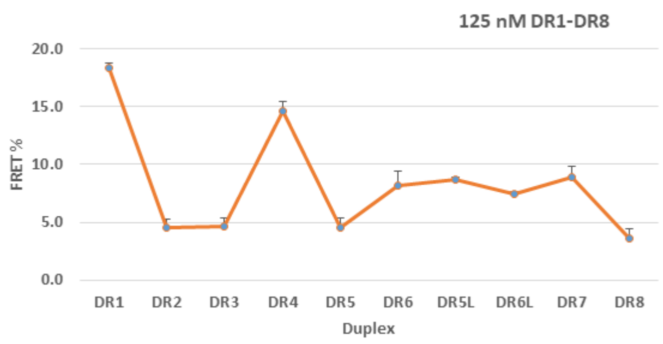

B)

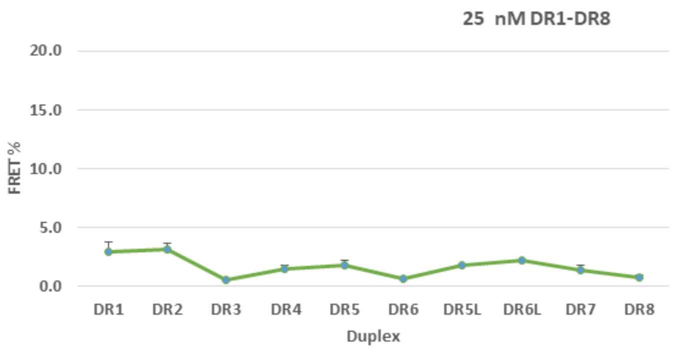
25 nM DR1-DR8
20.0
15.0
10.0
5.0
0.0
FRET %
DR1
DR2
DR3
DR4
DR5
DR6
DR5L
DR6L
DR7
DR8
Duplex

C)

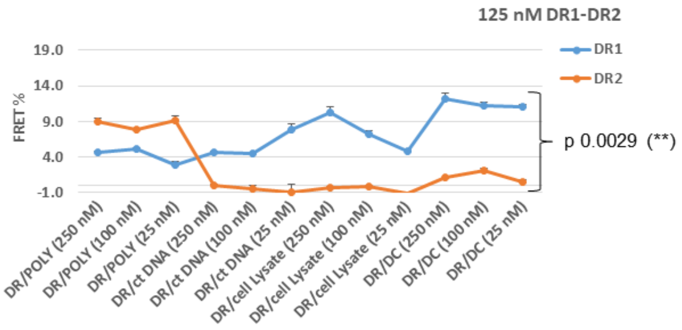
125 nM DR1-DR2
DR1
DR2
FRET %
19.0
14.0
9.0
4.0
-1.0
p 0.0029 (**)
DR/POLY (250 nM)
DR/POLY (100 nM)
DR/POLY (25 nM)
DR/ct DNA (250 nM)
DR/ct DNA (100 nM)
DR/ct DNA (25 nM)
DR/cell Lysate (250 nM)
DR/cell Lysate (100 nM)
DR/cell Lysate (25 nM)
DR/DC (250 nM)
DR/DC (100 nM)
DR/DC (25 nM)

D)

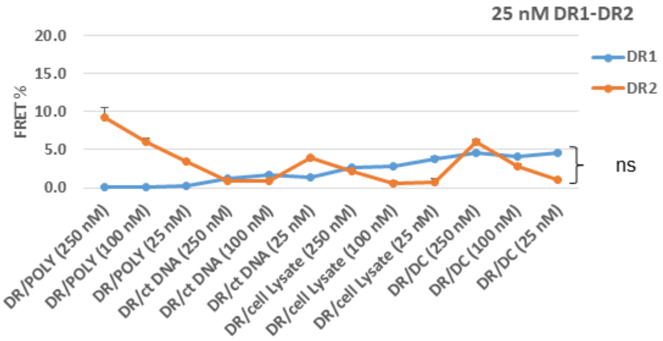
25 nM DR1-DR2
DR1
DR2
FRET %
20.0
15.0
10.0
5.0
0.0
ns
DR/POLY (250 nM)
DR/POLY (100 nM)
DR/POLY (25 nM)
DR/ct DNA (250 nM)
DR/ct DNA (100 nM)
DR/ct DNA (25 nM)
DR/cell Lysate (250 nM)
DR/cell Lysate (100 nM)
DR/cell Lysate (25 nM)
DR/DC (250 nM)
DR/DC (100 nM)
DR/DC (25 nM)

E)

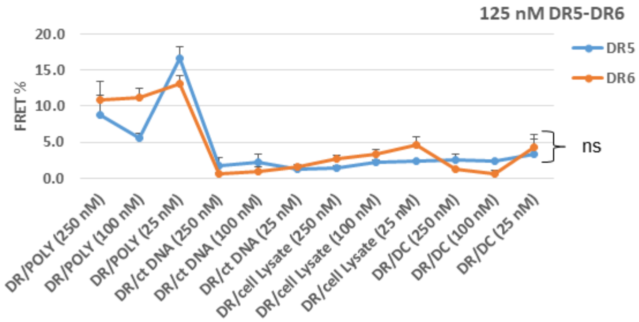
125 nM DR5-DR6
DR5
DR6
FRET %
20.0
15.0
10.0
5.0
0.0
ns
DR/POLY (250 nM)
DR/POLY (100 nM)
DR/POLY (25 nM)
DR/ct DNA (250 nM)
DR/ct DNA (100 nM)
DR/ct DNA (25 nM)
DR/cell Lysate (250 nM)
DR/cell Lysate (100 nM)
DR/cell Lysate (25 nM)
DR/DC (250 nM)
DR/DC (100 nM)
DR/DC (25 nM)

F)

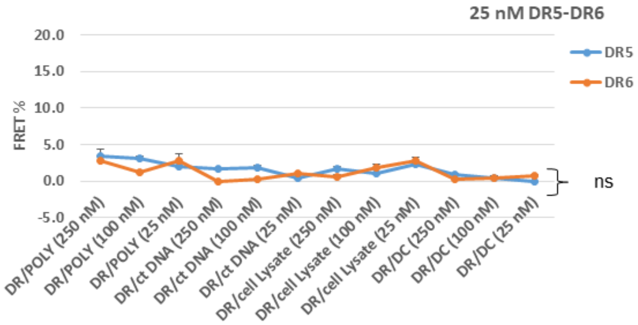
25 nM DR5-DR6
DR5
DR6
FRET %
20.0
15.0
10.0
5.0
0.0
-5.0
ns
DR/POLY (250 nM)
DR/POLY (100 nM)
DR/POLY (25 nM)
DR/ct DNA (250 nM)
DR/ct DNA (100 nM)
DR/ct DNA (25 nM)
DR/cell Lysate (250 nM)
DR/cell Lysate (100 nM)
DR/cell Lysate (25 nM)
DR/DC (250 nM)
DR/DC (100 nM)
DR/DC (25 nM)

Supplementary Figure 2. Representation of FRET efficiency values for tested duplexes DR5L-DR8 in the presence of additional reagents. POLY = poly-L-lysine; ct DNA = calf thymus DNA; DC = synthetic dsDNA crowder. ns = not significant (p>0.05); (*) = statistically significant with p < 0.05.

A)

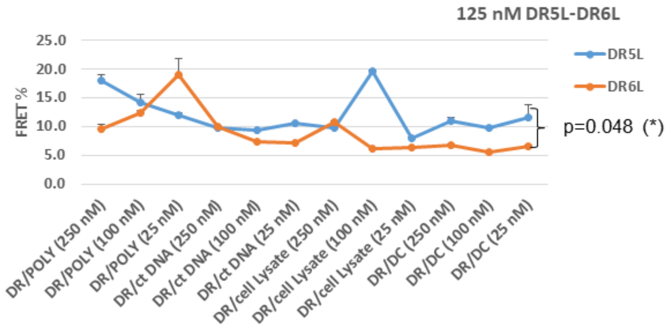

B)

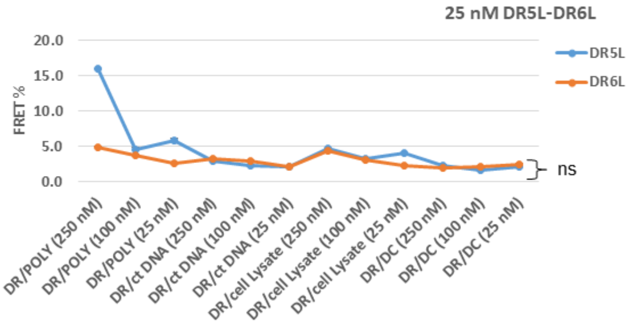

C)

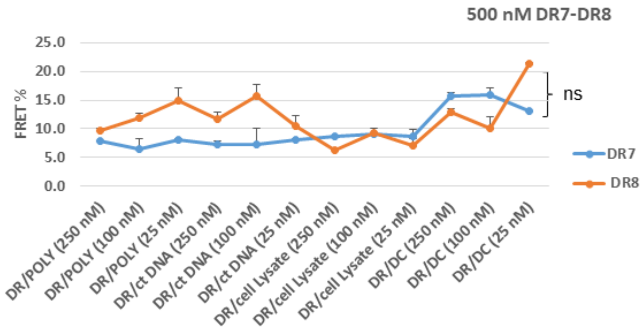

D)

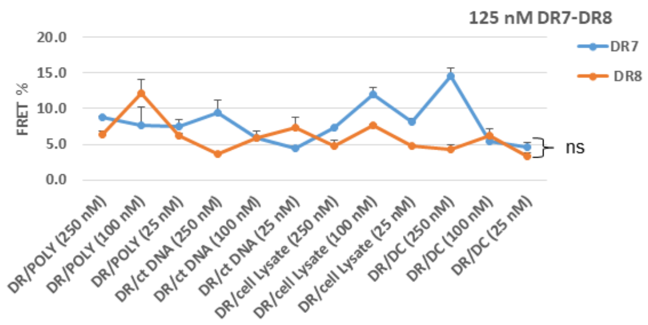
125 nM DR7-DR8
DR7
DR8
FRET %
20.0
15.0
10.0
5.0
0.0
ns
DR/POLY (250 nM)
DR/POLY (100 nM)
DR/POLY (25 nM)
DR/ct DNA (250 nM)
DR/ct DNA (100 nM)
DR/ct DNA (25 nM)
DR/cell Lysate (250 nM)
DR/cell Lysate (100 nM)
DR/cell Lysate (25 nM)
DR/DC (250 nM)
DR/DC (100 nM)
DR/DC (25 nM)

E)

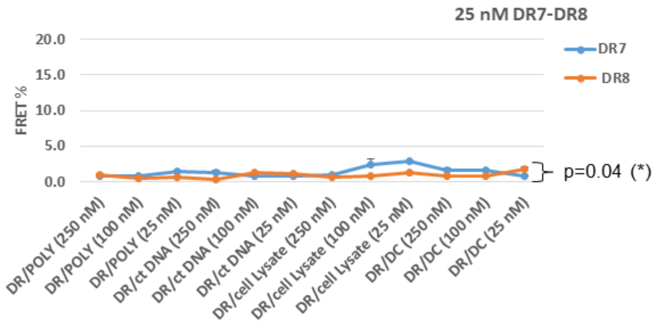
25 nM DR7-DR8
DR7
DR8
FRET %
20.0
15.0
10.0
5.0
0.0
p=0.04 (*)
DR/POLY (250 nM)
DR/POLY (100 nM)
DR/POLY (25 nM)
DR/ct DNA (250 nM)
DR/ct DNA (100 nM)
DR/ct DNA (25 nM)
DR/cell Lysate (250 nM)
DR/cell Lysate (100 nM)
DR/cell Lysate (25 nM)
DR/DC (250 nM)
DR/DC (100 nM)
DR/DC (25 nM)

Supplementary Figure 3. Representation of FRET efficiency values for tested duplexes DR3-DR4 in the presence of additional reagents. POLY = poly-L-lysine; ct DNA = calf thymus DNA; DC = synthetic dsDNA crowder. ns = not significant (p>0.05).

A)

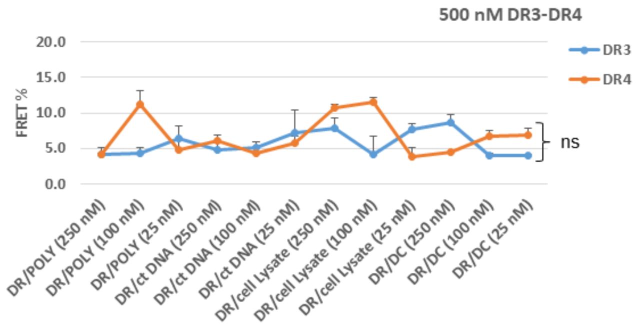

B)

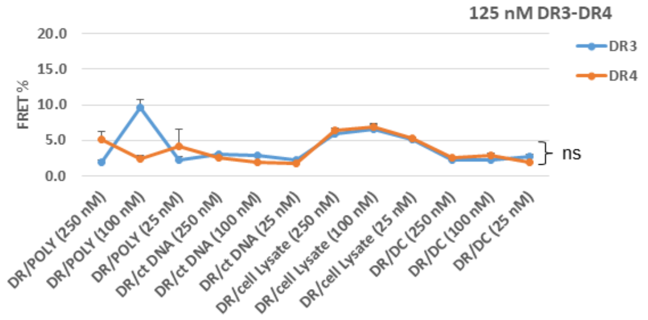

C)

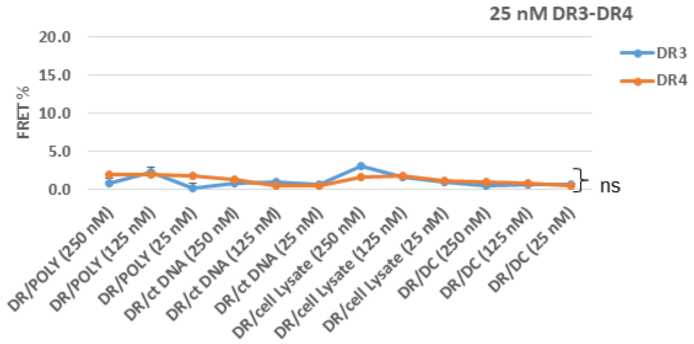

Tm studies

Supplementary Figure 4. Representation of FRET efficiency values for tested duplexes DR1-DR2, DR5-DR6, and DR5L-DR6L, at 25 nM concentration in the presence of PEG2000 (A), 2 nM leukemia cell mRNA (B). Correlation between T_m and the distances Donor-Acceptor determined by FRET, in duplex D1 at 1000 nM (C) and 500 nM (D) concentrations. DC = duplex control; ct DNA = calf thymus DNA. Each measurement was performed twice (n=2) and the error values were calculated.

A)

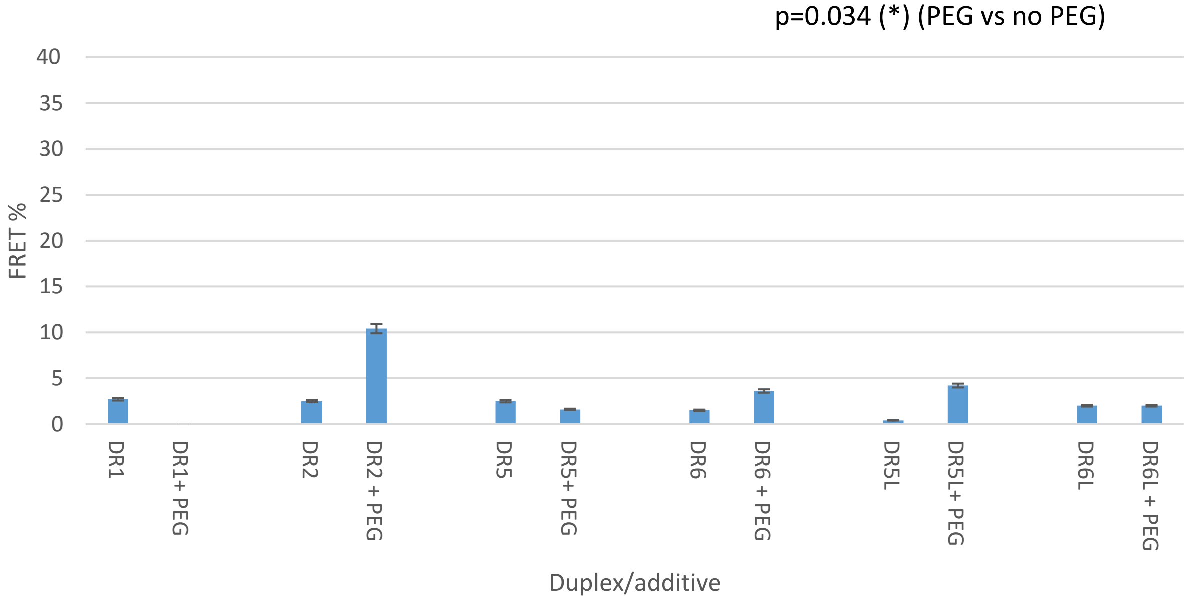

B)

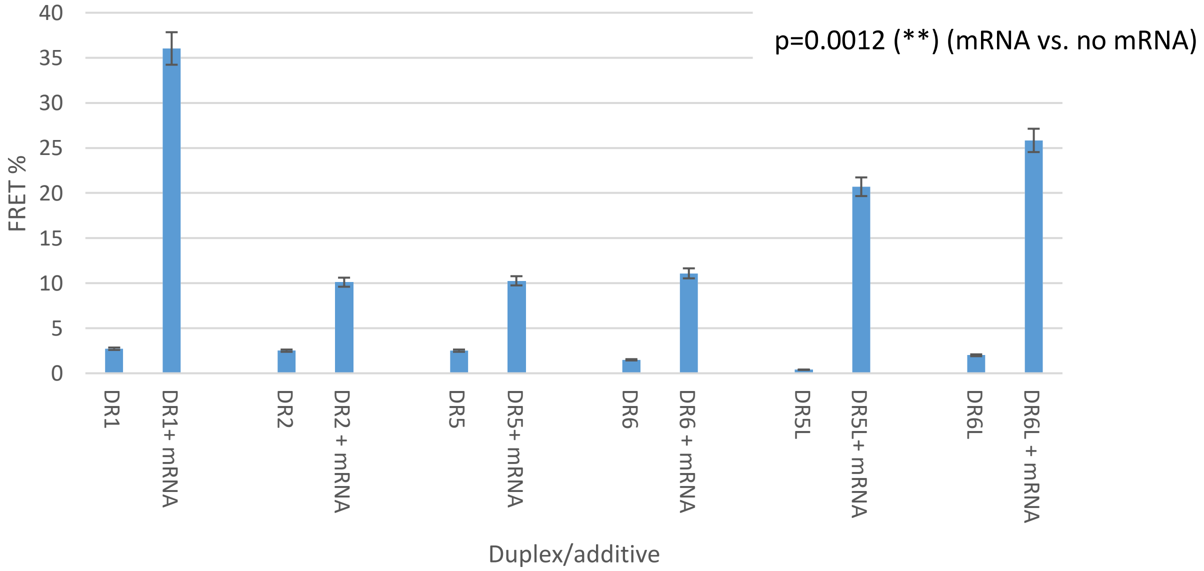

C)

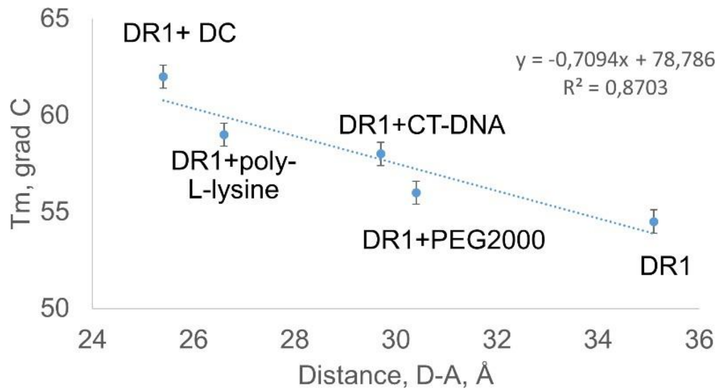
DR1+ DC
y = -0,7094x + 78,786
R² = 0,8703
DR1+CT-DNA
DR1+poly-
L-lysine
DR1+PEG2000
DR1
Tm, grad C
Distance, D-A, Å
65
60
55
50
24
26
28
30
32
34
36

D)

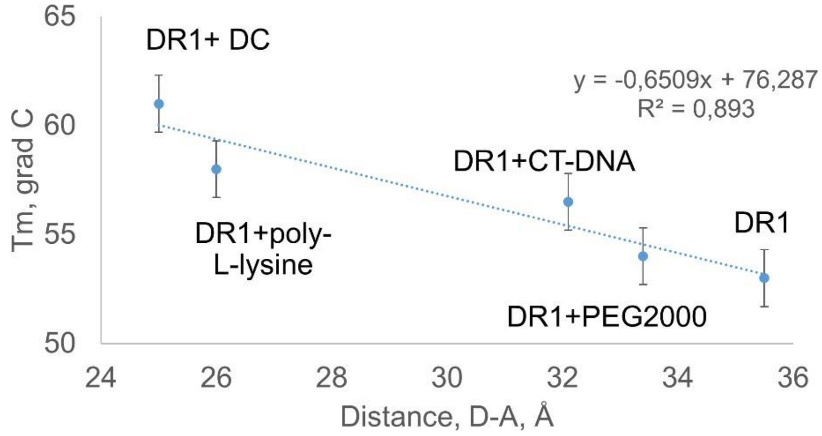
DR1+ DC
y = -0,6509x + 76,287
R² = 0,893
DR1+CT-DNA
DR1
DR1+poly-
L-lysine
DR1+PEG2000
Tm, grad C
Distance, D-A, Å
65
60
55
50
24
26
28
30
32
34
36

Supplementary Table 3. T_m values for DR1 and DR1-CTRL at different concentrations.

				T_m, °C			
**Duplex/Concentration**	**500 nM**	**400 nM**	**250 nM**	**125 nM**	**50 nM**	**25 nM**	**5 nM**
DR1	58.5	57.5	57.5	57.5	nct*	nct	nct
DR1-CTRL	57.0	54.5	54.5	54	nct	nct	nct

* nct = no clear transition.

Supplementary Figure 5. Representative thermal denaturation curves for DR1-CTRL (blue) and DR1 (orange) at different concentrations in 1X PBS, pH 7.2.

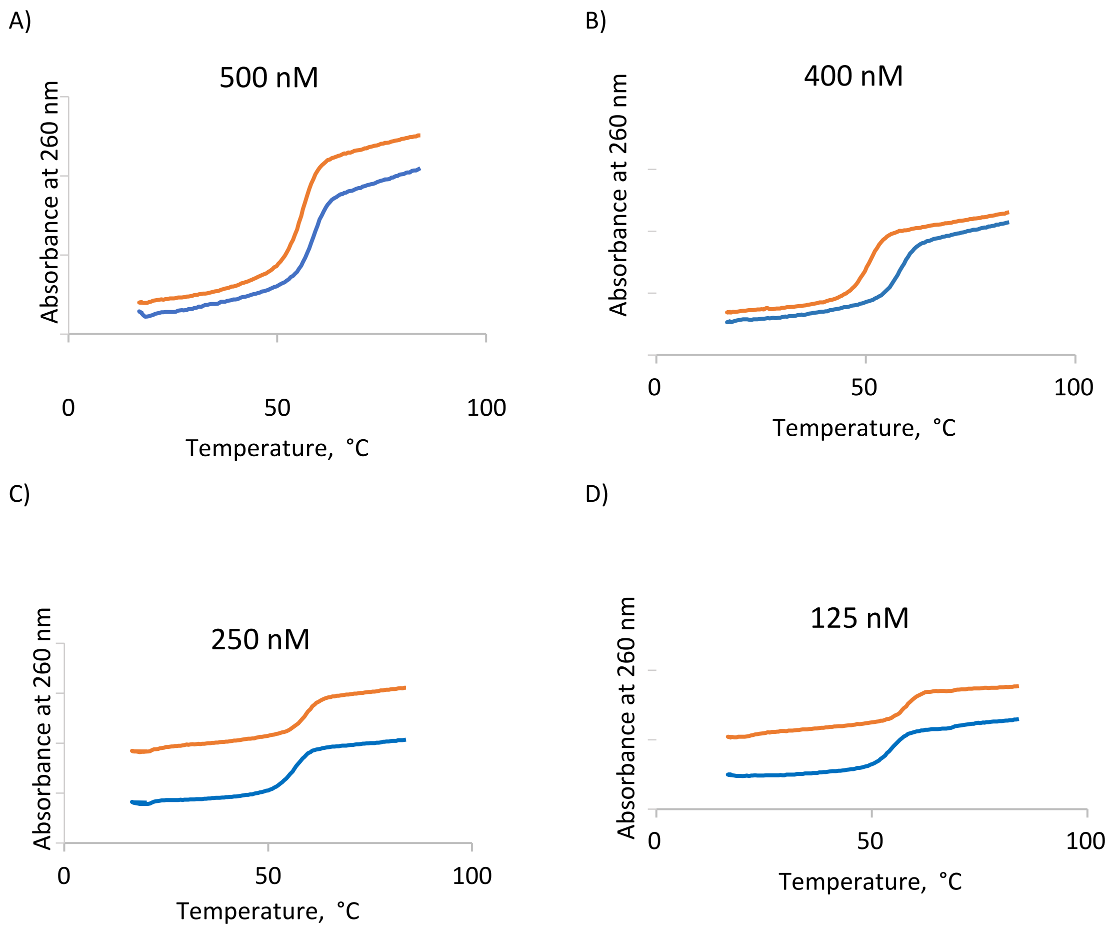

Supplementary Figure 6. Thermal denaturation curves for DR1 (1 µM) + DC (grey), DR1-CTRL (1 µM) + DC (yellow), DR1 (250 nM) + DC (blue), and DR1-CTRL (250 nM) + DC (green). DC = synthetic dsDNA crowder.

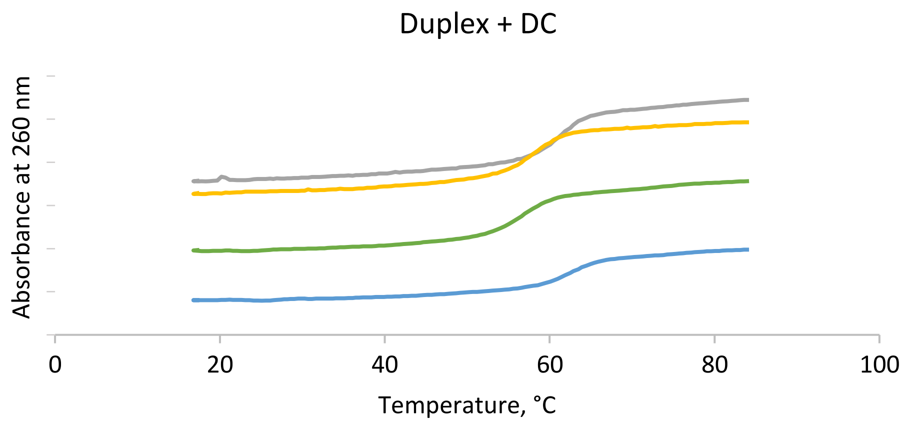

Supplementary Figure 7. Thermal denaturation curves for DR1 (1 µM) + ct DNA (grey), DR1- CTRL (1 µM) + ct DNA (yellow), DR1 (250 nM) + ct DNA (blue), and DR1-CTRL (250 nM) + ct DNA (green). Ct DNA = calf thymus DNA

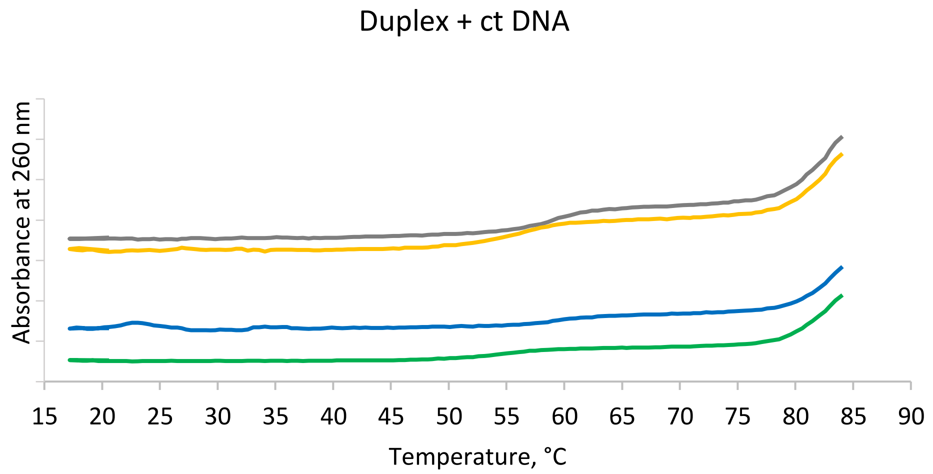

.

Supplementary Figure 8. Thermal denaturation curves for DR1 (1 µM) + PEG (grey), DR1-CTRL (1 µM) + PEG (yellow), DR1 (250 nM) + PEG (blue), and DR1-CTRL (250 nM) + PEG (green)

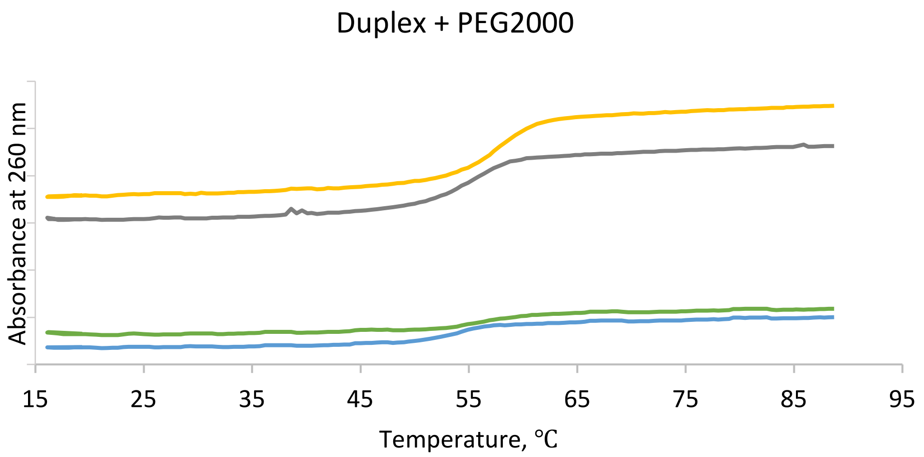

Supplementary Table 4. T_m data for DR1 and DR1-CTRL in presence of additive reagents.

Reagent/Duplex (concentration)	DR1	DR1-CTRL
	T_m, °C	
Poly-L-lysine (100 nM)/ duplex (1000 nM)	59.0	57.0
Poly-L-lysine (100 nM)/ duplex (250 nM)		
	59.0	58.0
ct DNA (2.5 nM)/ duplex (1000 nM)		
first transition of T_m graph	58.0	57.0
ct DNA (2.5 nM)/ duplex (250 nM)		
first transition of T_m graph	57.0	54.0
ct DNA (2.5 nM)/ duplex (1000 nM)		
second transition of T_m graph	83.5	83.0
ct DNA (2.5 nM)/ duplex (250 nM)		
second transition of T_m graph		
	83.0	80.0
DC (100 nM)/ duplex (1000 nM)	62.0	62.0
DC (100 nM)/ duplex (250 nM)		
	57.0	57.0
PEG2000 (100 nM)/ duplex (1000 nM)	56.0	55.0
PEG2000 (100 nM)/ duplex (250 nM)	55.0	54.0

ct DNA = calf thymus DNA; DC = synthetic dsDNA crowder.

CD measurements

Supplementary Figure 9. Representative CD curves for the duplexes DR1 (orange) and DR1-CTRL (blue) in different media. DC = synthetic dsDNA crowder ; ct DNA = calf thymus DNA.

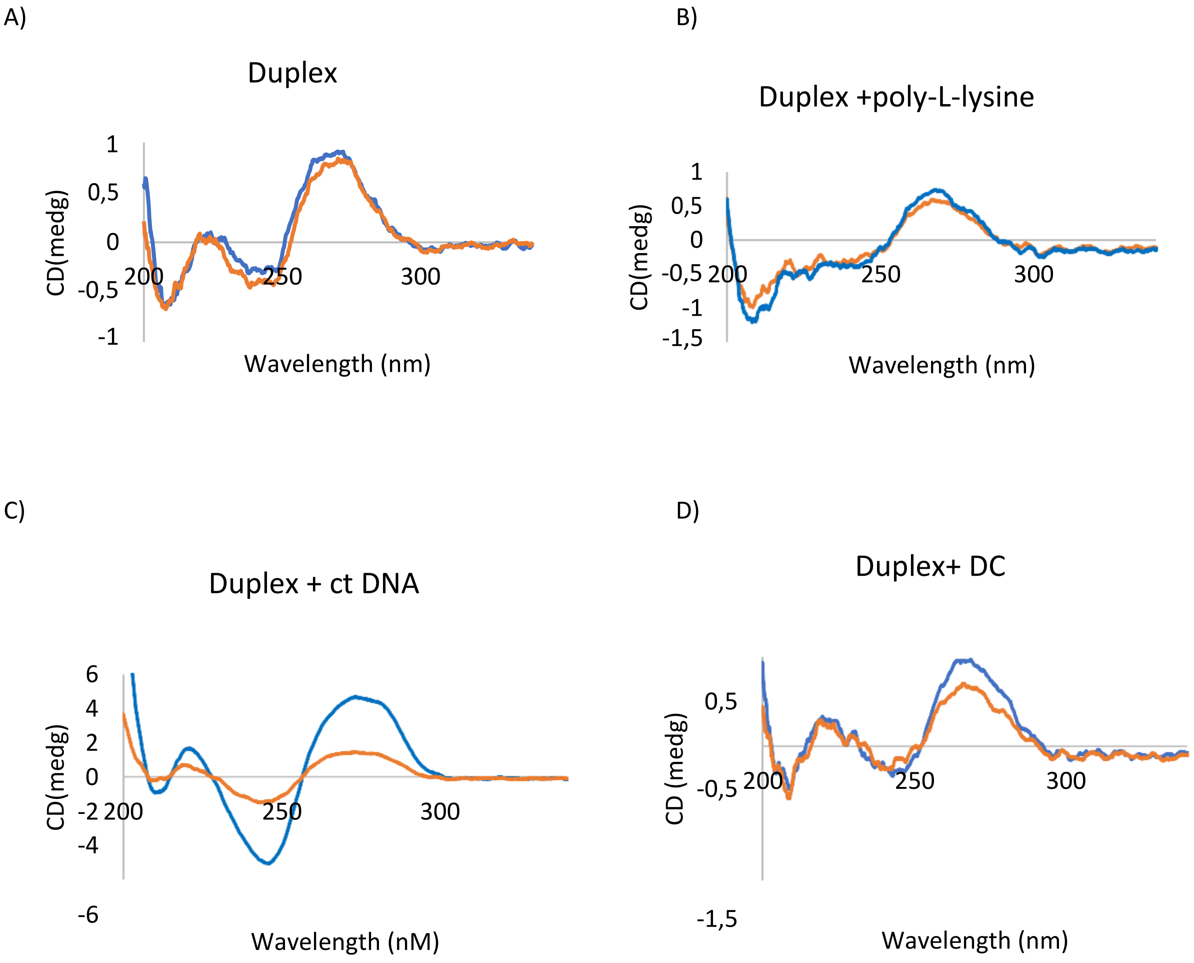

E)

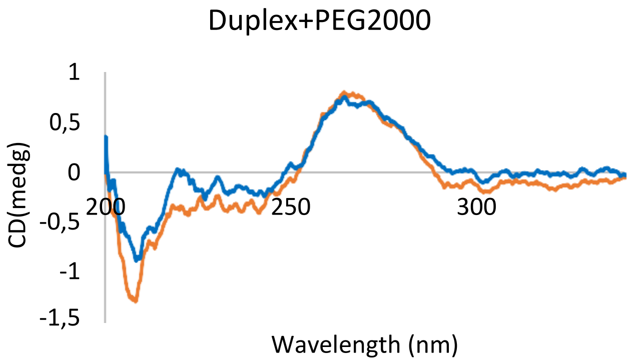

Supplementary Note 1. Parameter optimization

Mesoscopic modelling was used to extract oligonucleotide parameters from the measured duplex melting temperatures, following the procedures outlined elsewhere.[2,3] Here we will briefly outline the main equations and introduce the relevant parameters.

The mesoscopic Peyrard-Bishop (PB) model uses two potentials, one for describing the hydrogen bonds and another for the stacking interactions.[4] The hydrogen bonds for the i-th base pair are represented by a Morse potential[5]

$$V(y_i) = D_\alpha \left(e^{-y_i/\lambda_\alpha} - 1\right)^2 \tag{1}$$

where D_α is the potential depth and λ_α the potential width for a base pair of type α. The variable y is the relative displacement between the bases, that is, the difference between the displacements of each strand that contributes to the stretching of the hydrogen bonds. The stacking interaction between the i-th and $i+1$-th base pairs is given by

$$w(y_i, y_{i+1}) = \frac{k_\beta}{2}\left(y_i^2 - 2y_i y_{i+1}\cos\theta + y_{i+1}^2\right), \tag{2}$$

where k_β is elastic constant of a nearest-neighbour of type β.

The partition function (see for instance Eq. (14) of Ref. 6) is integrated over 400 points in the interval $y_{\text{min}} = -0.1$ nm to $y_{\text{max}} = 20.0$ nm, and a eigenvalue cut-off of 10, see Eq. (22) of Ref. 6. The adimensional thermal index τ is calculated from the partition function[7] and correlated to the melting temperatures using a linear regression

$$T'_n(P) = a_0 + a_1\tau_n(P), \tag{3}$$

where T'_i is the predicted temperature for the n-th sequence in the dataset and P is the tentative set of model parameters. The regression coefficients a_0,a_1 are obtained from the experimental melting temperatures. The optimization process consists in minimizing the merit function

$$\chi_j^2 = \sum_{n=1}^{N}\left[T'_n(P_j) - T_n\right]^2. \tag{4}$$

where T_n is the calculated melting temperature of the n-th, and P_j is the j-th tentative parameter set and N is the number of melting temperatures in the dataset. Eq. (4) is numerically minimized with the downhill simplex method[8] and to overcome the well known problem o local minima, this minimization is repeated several times starting over with different initial parameters following the procedures outlined in Refs. 2,6,9. The final parameters shown here are those for the lowest overall χ^2, and we estimate the parameter uncertainty from the standard deviations over all minimizations.

Another quality parameter of interest is average difference between the experimental and the calculated temperatures

$$\langle \Delta T\rangle = \frac{1}{N}\sum_{n=1}^{N}\left|T'_n - T_n\right|. \tag{5}$$

Further details on the model implementation are given in Refs. 2,6,9.

For some sequences we calculated the average base pair displacement $\langle y_i\rangle$ which is obtained from the partition function using the potentials of Eq. (1) and (2). This is based on the method developed by Zhang et al.[10], for implementation details on this type of calculation see Ref. 9. The uncertainly of the average base pair displacement is estimated by randomly changing (normal distribution) the parameters within their standard veriations and recalculating $\langle y_i\rangle$. This is repeated

100 times for each sequence and is shown in Figs 3–5 as error bars. Note that for most cases the resulting error bars are too small to be visible.

For the parametrization, we will vary only the Morse potential depth D and the stacking elastic constant k, following the procedure developed previously.[2,3] The Morse potential width λ and the angle θ are kept constant during the optimization.

1.1 DNA/LNA:DNA optimization

Here, due to the large number of unknown parameters the process was split into a number separate steps to ultimately arrive at hydrogen bonding and stacking information about the LNA modified probes. The strategy was to split the dataset into sequences with and without LNA modified bases. We worked out all the parameters in the absence of LNA, even though substantial uncertainty was to be expected. We then kept these parameters constant, and in a final step optimized solely LNA modifications.

1.1.1 Data set description

The sequences had mixed characteristics, canonical duplex DNA, some had attached fluorophores, some with overhangs and some were LNA modified. All sequences and their respective melting temperatures are shown in supplementary table 5. For the purpose of the parameter optimization they were divided into the following data sets,

Set F All duplexes without LNA modifications, code notation for overhangs and fluorophores are shown in Tab. 6 and 7, respectively. For the purpose of the parametrization, Set F was subdivided into the following subsets:

Subset C strictly canonical DNA duplexes, no overhangs or fluorophores

Subset O duplexes with and without overhangs, but no fluorophores

Set L only duplexes containing LNA modifications. The overhangs and fluorophores are the same as in Set F, therefore we maintain the notations of Tabs. 6 and 7

1.1.2 Parametrization workflow

Step 1 Parametrization of Subset C, using previously calculated DNA parameters at [Na^{+}] 69 mM from Ref. 2 as initial parameters, we arrived at preliminary parameters for AT and CG.

Step 2 Using the preliminary parameters of Step 1, we performed the Parametrization of Subset O. We obtained the Morse potentials for the overhangs, see columns labelled 'overhang' in Tab. 8. We also worked the perturbation of the overhangs on the first neighbouring base-pair, see base-pairs in notation BP^{XV} in Tab. 8. For the canonical DNA base-pairs the optimized Morse potentials are shown in the first row of Tab. 8 and the new stacking interaction parameters are shown in Tab. 9.

Step 3 We now keep the parameters obtained in Step 2 as constants, and parametrize Set F containing the fluorophores. For this we assign the fluorophore notation shown in Tab. 7, following a similar procedure as previously used for Cy3/Cy5 dyes.[11] As in Step 2, we allow the variation of Morse potentials of base-pairs next to the fluorophores, these results are shown in Tab. 10, where the we also show the potentials associated to fluorophores in the absence of overhangs. Table 11 shows the potentials when the fluorophores are paired with overhangs. The stacking interaction parameters for all these cases are shown in Tab. 12.

Step 4 Many of the sequences in Set L have similar counterparts in Set F, that is, most of Set L is similar to set F except for the presence of LNA modified nucleotides, including similar overhangs and fluorophores. Therefore, we now keep all parameters of Steps 2 and 3 constant, Tabs. 8–12, and vary only the Morse and stacking potentials of the LNA modified bases. The final results for LNA modified bases are shown in Tab. 13 and 14.

Supplementary Table 5: Sequences for the model probes of type DNA/DNA (DD) and their measured and predicted melting temperatures, T_{exp} and T_{pre}, respectively, at total strand concentrations 0.3, 0.6 and 1.0 μM. All temperatures are in °C. In LNA containing sequences, the positions of LNA nucleotides are indicated in red. Overhangs in target DNA with regard to the probe binding region are shown in blue. Fluorophores are shown in green. Main DNA strands are shown in 5′ →3′ on top of complementary DNA strands in 3′ ←5′ direction. The final quality parameters of the optimization were $\chi^2 = 214\,°\text{C}^2$ and $\langle \Delta T \rangle = 1.3\,°\text{C}$.

identification	Duplex	1.0 μM T_{exp}	1.0 μM T_{pre}	0.6 μM T_{exp}	0.6 μM T_{pre}	0.3 μM T_{exp}	0.3 μM T_{pre}
DD17-CTRL	GAG CGG ATG GCG TAG GCA<br>CTC GCC TAC CGC ATC CGT	68.0	68.3	—	—	68.5	70.1
DD18-CTRL	GAG AGT CAG TCA GTC TGT<br>CTC TCA GTC AGT CAG ACA	61.6	61.3	—	—	—	—
DD19-CTRL	AGA GAT TTC TCT GTA GTT<br>TCT CTA AAG AGA CAT CAA	54.5	54.5	—	—	—	—
DD17-CTRL′	GAG CGG ATG GCG TAG GCA<br>ACACCATCAACTC GCC TAC CGC ATC CGT	67.5	67.6	—	—	—	—
DD17-CTRL″	GAG CGG ATG GCG TAG GCA<br>ACACCCTC GCC TAC CGC ATC CGTATCAA	68.0	67.9	—	—	70.2	70.1
DD18-CTRL′	GAG AGT CAG TCA GTC TGT<br>AGAAAGGAGACTC TCA GTC AGT CAG ACA	60.2	60.1	—	—	—	—
DD18-CTRL″	GAG AGT CAG TCA GTC TGT<br>AGAAACTC TCA GTC AGT CAG ACAGGAGA	60.6	60.4	—	—	—	—
DD19-CTRL′	AGA GAT TTC TCT GTA GTT<br>AGGTGAGGTATCT CTA AAG AGA CAT CAA	52.5	52.7	—	—	—	—
DD19-CTRL″	AGA GAT TTC TCT GTA GTT<br>AGGTGTCT CTA AAG AGA CAT CAAAGGTA	52.5	52.5	—	—	—	—
DD17a	/Cy3/GAG CGG ATG GCG TAG GCA<br>CTC GCC TAC CGC ATC CGT	52.2	55.6	52.0	54.4	51.2	53.5
DD17a′	/Cy3/GAG CGG ATG GCG TAG GCA<br>ACACCATCAACTC GCC TAC CGC ATC CGT	52.3	56.4	52.0	55.4	51.0	54.4
DD17a″	/Cy3/GAG CGG ATG GCG TAG GCA<br>ACACCCTC GCC TAC CGC ATC CGTATCAA	65.2	62.6	65.0	63.2	64.0	62.0
DD18a	/Cy3/GAG AGT CAG TCA GTC TGT<br>CTC TCA GTC AGT CAG ACA	55.7	52.0	52.0	50.0	51.5	49.1
DD18a′	/Cy3/GAG AGT CAG TCA GTC TGT<br>AGAAAGGAGACTC TCA GTC AGT CAG ACA	55.0	53.9	53.0	52.3	51.0	51.4
DD18a″	/Cy3/GAG AGT CAG TCA GTC TGT<br>AGAAACTC TCA GTC AGT CAG ACAGGAGA	55.5	53.7	53.5	52.1	50.5	51.1
DD19a	/Cy3/AGA GAT TTC TCT GTA GTT<br>TCT CTA AAG AGA CAT CAA	52.7	53.4	52.3	51.7	52.0	50.8
DD19a′	/Cy3/AGA GAT TTC TCT GTA GTT<br>AGGTGAGGTATCT CTA AAG AGA CAT CAA	53.2	53.8	52.7	52.2	52.0	51.3
DD19a″	/Cy3/AGA GAT TTC TCT GTA GTT<br>AGGTGTCT CTA AAG AGA CAT CAAAGGTA	52.8	53.2	50.2	51.4	51.0	50.5

(continued on next page)

(5 continued)

identification	Duplex	1.0 μM T_{exp}	1.0 μM T_{pre}	0.6 μM T_{exp}	0.6 μM T_{pre}	0.3 μM T_{exp}	0.3 μM T_{pre}
DD17b	/Cy5/GAG CGG ATG GCG TAG GCA<br>CTC GCC TAC CGC ATC CGT	49.9	54.3	50.0	54.0	49.0	53.3
DD17b’	/Cy5/GAG CGG ATG GCG TAG GCA<br>ACACCATCAACTC GCC TAC CGC ATC CGT	54.3	54.4	54.0	54.0	53.0	53.3
DD17b”	/Cy5/GAG CGG ATG GCG TAG GCA<br>ACACCCTC GCC TAC CGC ATC CGTATCAA	55.6	54.4	55.3	54.0	54.8	53.4
DD18b	/Cy5/GAG AGT CAG TCA GTC TGT<br>CTC TCA GTC AGT CAG ACA	54.0	53.9	52.0	53.4	51.5	52.5
DD18b’	/Cy5/GAG AGT CAG TCA GTC TGT<br>AGAAAGGAGACTC TCA GTC AGT CAG ACA	55.6	53.9	55.3	53.4	54.3	52.5
DD18b”	/Cy5/GAG AGT CAG TCA GTC TGT<br>AGAAACTC TCA GTC AGT CAG ACAGGAGA	57.3	54.0	57.0	53.5	56.6	52.6
DD19b	/Cy5/AGA GAT TTC TCT GTA GTT<br>TCT CTA AAG AGA CAT CAA	53.6	53.3	52.7	52.7	51.2	51.4
DD19b’	/Cy5/AGA GAT TTC TCT GTA GTT<br>AGGTGAGGTATCT CTA AAG AGA CAT CAA	52.7	53.3	52.3	52.7	51.5	51.5
DD19b”	/Cy5/AGA GAT TTC TCT GTA GTT<br>AGGTGTCT CTA AAG AGA CAT CAAAGGTA	51.7	53.3	51.8	52.7	50.0	51.4
DD17c	/ATTO532/GAG CGG ATG GCG TAG GCA<br>CTC GCC TAC CGC ATC CGT	68.0	67.9	68.3	67.6	66.0	65.5
DD17c’	/ATTO532/GAG CGG ATG GCG TAG GCA<br>ACACCATCAACTC GCC TAC CGC ATC CGT	69.5	68.1	68.3	67.8	66.5	65.7
DD17c”	/ATTO532/GAG CGG ATG GCG TAG GCA<br>ACACCCTC GCC TAC CGC ATC CGTATCAA	67.5	68.1	68.3	67.8	65.0	65.7
DD18c	/ATTO532/GAG AGT CAG TCA GTC TGT<br>CTC TCA GTC AGT CAG ACA	62.6	62.8	61.4	62.4	61.0	60.9
DD18c’	/ATTO532/GAG AGT CAG TCA GTC TGT<br>AGAAAGGAGACTC TCA GTC AGT CAG ACA	62.8	63.6	62.5	63.3	61.0	61.7
DD18c”	/ATTO532/GAG AGT CAG TCA GTC TGT<br>AGAAACTC TCA GTC AGT CAG ACAGGAGA	63.3	63.9	62.9	63.6	61.5	61.9
DD19c	/ATTO532/AGA GAT TTC TCT GTA GTT<br>TCT CTA AAG AGA CAT CAA	45.0	45.3	44.7	44.8	44.0	45.2
DD19c’	/ATTO532/AGA GAT TTC TCT GTA GTT<br>AGGTGAGGTATCT CTA AAG AGA CAT CAA	46.1	46.2	45.6	45.7	47.5	46.0
DD19c”	/ATTO532/AGA GAT TTC TCT GTA GTT<br>AGGTGTCT CTA AAG AGA CAT CAAAGGTA	54.0	52.9	53.5	52.4	52.0	52.0
DD17d	/ATTO647/GAG CGG ATG GCG TAG GCA<br>CTC GCC TAC CGC ATC CGT	67.2	68.8	68.0	70.6	66.9	69.0
DD17d’	/ATTO647/GAG CGG ATG GCG TAG GCA<br>ACACCATCAACTC GCC TAC CGC ATC CGT	68.0	68.5	71.0	70.3	67.9	68.8
DD17d”	/ATTO647/GAG CGG ATG GCG TAG GCA<br>ACACCCTC GCC TAC CGC ATC CGTATCAA	68.5	67.4	68.8	68.8	67.4	67.4
DD18d	/ATTO647/GAG AGT CAG TCA GTC TGT<br>CTC TCA GTC AGT CAG ACA	62.0	60.9	61.3	60.6	60.0	59.9
DD18d’	/ATTO647/GAG AGT CAG TCA GTC TGT<br>AGAAAGGAGACTC TCA GTC AGT CAG ACA	62.0	61.9	62.8	61.8	63.4	61.0
DD18d”	/ATTO647/GAG AGT CAG TCA GTC TGT<br>AGAAACTC TCA GTC AGT CAG ACAGGAGA	64.0	62.7	63.6	62.8	63.9	61.9
DD19d	/ATTO647/AGA GAT TTC TCT GTA GTT<br>TCT CTA AAG AGA CAT CAA	54.1	55.1	54.0	53.2	53.0	53.1
DD19d’	/ATTO647/AGA GAT TTC TCT GTA GTT<br>AGGTGAGGTATCT CTA AAG AGA CAT CAA	48.7	48.5	43.4	44.7	44.0	45.4

(continued on next page)

(5 continued)

identification	Duplex	1.0 µM T_{exp}	1.0 µM T_{pre}	0.6 µM T_{exp}	0.6 µM T_{pre}	0.3 µM T_{exp}	0.3 µM T_{pre}
DD19d”	/ATTO647/AGA GAT TTC TCT GTA GTT<br>AGGTGTCT CTA AAG AGA CAT CAAAGGTA	55.1	55.9	54.0	54.2	54.0	54.0
DD17L-CTRL	GAG CGG AT+G +G+CG TAG GCA<br>CTC GCC TAC CGC ATC CGT	—	—	—	—	72.5	70.1
DD17L1-CTRL”	GAG CGG AT+G +G+CG TAG GCA<br>ACACC CTC GCC TAC CGC ATC CGT ATCAA	—	—	—	—	74.0	70.1
DD17L2-CTRL	GAG CGG AT+G GCG TAG GCA<br>CTC GCC TAC CGC ATC CGT	—	—	—	—	67.0	70.0
DD17L2-CTRL”	GAG CGG AT+G GCG TAG GCA<br>ACACC CTC GCC TAC CGC ATC CGT ATCAA	—	—	—	—	68.4	70.0
DD17L3-CTRL	GAG CGG ATG +GCG TAG GCA<br>CTC GCC TAC CGC ATC CGT	—	—	—	—	67.3	70.1
DD17L3-CTRL”	GAG CGG ATG +GCG TAG GCA<br>ACACC CTC GCC TAC CGC ATC CGT ATCAA	—	—	—	—	69.0	70.1
DD17L4-CTRL	GAG CGG ATG G+CG TAG GCA<br>CTC GCC TAC CGC ATC CGT	—	—	—	—	68.0	70.2
DD17L4-CTRL”	GAG CGG ATG G+CG TAG GCA<br>ACACC CTC GCC TAC CGC ATC CGT ATCAA	—	—	—	—	70.1	70.2
DD17L5-CTRL	GAG CGG AT+G +GCG TAG GCA<br>CTC GCC TAC CGC ATC CGT	—	—	—	—	70.6	70.1
DD17L5-CTRL”	GAG CGG AT+G +GCG TAG GCA<br>ACACC CTC GCC TAC CGC ATC CGT ATCAA	—	—	—	—	72.1	70.1
DD17L6-CTRL	GA+G CGG AT+G GCG TAG GCA<br>CTC GCC TAC CGC ATC CGT	—	—	—	—	70.0	69.9
DD17L6-CTRL”	GA+G CGG AT+G GCG TAG GCA<br>ACACC CTC GCC TAC CGC ATC CGT ATCAA	—	—	—	—	71.8	69.9
DD17L7-CTRL	GA+G CGG ATG GCG TAG +GCA<br>CTC GCC TAC CGC ATC CGT	—	—	—	—	69.9	70.0
DD17L7-CTRL”	GA+G CGG ATG GCG TAG +GCA<br>ACACC CTC GCC TAC CGC ATC CGT ATCAA	—	—	—	—	71.5	70.0
DD17a-L1	/Cy3/GAG CGG AT+G +G+CG TAG GCA<br>CTC GCC TAC CGC ATC CGT	—	—	—	—	73.4	70.1
DD17a-L1”	/Cy3/GAG CGG AT+G +G+CG TAG GCA<br>ACACC CTC GCC TAC CGC ATC CGT ATCAA	—	—	—	—	77.2	73.6
DD17a-L2	/Cy3/GAG CGG AT+G GCG TAG GCA<br>CTC GCC TAC CGC ATC CGT	—	—	—	—	67.6	67.0
DD17a-L2”	/Cy3/GAG CGG AT+G GCG TAG GCA<br>ACACC CTC GCC TAC CGC ATC CGT ATCAA	—	—	—	—	69.0	70.8
DD17a-L3	/Cy3/GAG CGG ATG +GCG TAG GCA<br>CTC GCC TAC CGC ATC CGT	—	—	—	—	68.4	68.5
DD17a-L3”	/Cy3/GAG CGG ATG +GCG TAG GCA<br>ACACC CTC GCC TAC CGC ATC CGT ATCAA	—	—	—	—	70.3	72.0
DD17a-L4	/Cy3/GAG CGG ATG G+CG TAG GCA<br>CTC GCC TAC CGC ATC CGT	—	—	—	—	69.2	68.7
DD17a-L4”	/Cy3/GAG CGG ATG G+CG TAG GCA<br>ACACC CTC GCC TAC CGC ATC CGT ATCAA	—	—	—	—	71.0	73.6
DD17a-L5	/Cy3/GAG CGG AT+G +GCG TAG GCA<br>CTC GCC TAC CGC ATC CGT	—	—	—	—	71.2	69.5
DD17a-L5”	/Cy3/GAG CGG AT+G +GCG TAG GCA<br>ACACC CTC GCC TAC CGC ATC CGT ATCAA	—	—	—	—	75.0	72.8
DD17a-L6	/Cy3/GA+G CGG AT+G GCG TAG GCA<br>CTC GCC TAC CGC ATC CGT	—	—	—	—	66.0	66.2

(continued on next page)

(5 continued)

identification	Duplex	1.0 μM T_{exp}	1.0 μM T_{pre}	0.6 μM T_{exp}	0.6 μM T_{pre}	0.3 μM T_{exp}	0.3 μM T_{pre}
DD17a-L6”	/Cy3/GA+G CGG AT+G GCG TAG GCA<br>ACACC CTC GCC TAC CGC ATC CGT ATCAA	—	—	—	—	68.1	69.6
DD17a-L7	/Cy3/GA+G CGG ATG GCG TAG +GCA<br>CTC GCC TAC CGC ATC CGT	—	—	—	—	67.1	67.9
DD17a-L7”	/Cy3/GA+G CGG ATG GCG TAG +GCA<br>ACACC CTC GCC TAC CGC ATC CGT ATCAA	—	—	—	—	68.7	71.8
DD17b-L1	/Cy5/GAG CGG AT+G +G+CG TAG GCA<br>CTC GCC TAC CGC ATC CGT	—	—	—	—	68.2	68.2
DD17b-L1”	/Cy5/GAG CGG AT+G +G+CG TAG GCA<br>ACACC CTC GCC TAC CGC ATC CGT ATCAA	—	—	—	—	70.1	68.2
DD17b-L2	/Cy5/GAG CGG AT+G GCG TAG GCA<br>CTC GCC TAC CGC ATC CGT	—	—	—	—	63.2	64.1
DD17b-L2”	/Cy5/GAG CGG AT+G GCG TAG GCA<br>ACACC CTC GCC TAC CGC ATC CGT ATCAA	—	—	—	—	64.2	65.1
DD17b-L3	/Cy5/GAG CGG ATG +GCG TAG GCA<br>CTC GCC TAC CGC ATC CGT	—	—	—	—	66.1	66.5
DD17b-L3”	/Cy5/GAG CGG ATG +GCG TAG GCA<br>ACACC CTC GCC TAC CGC ATC CGT ATCAA	—	—	—	—	67.4	67.3
DD17b-L4	/Cy5/GAG CGG ATG G+CG TAG GCA<br>CTC GCC TAC CGC ATC CGT	—	—	—	—	67.1	67.0
DD17b-L4”	/Cy5/GAG CGG ATG G+CG TAG GCA<br>ACACC CTC GCC TAC CGC ATC CGT ATCAA	—	—	—	—	68.9	68.6
DD17b-L5	/Cy5/GAG CGG AT+G +GCG TAG GCA<br>CTC GCC TAC CGC ATC CGT	—	—	—	—	67.3	68.3
DD17b-L5”	/Cy5/GAG CGG AT+G +GCG TAG GCA<br>ACACC CTC GCC TAC CGC ATC CGT ATCAA	—	—	—	—	68.4	69.1
DD17b-L6	/Cy5/GA+G CGG AT+G GCG TAG GCA<br>CTC GCC TAC CGC ATC CGT	—	—	—	—	63.2	63.7
DD17b-L6”	/Cy5/GA+G CGG AT+G GCG TAG GCA<br>ACACC CTC GCC TAC CGC ATC CGT ATCAA	—	—	—	—	65.7	63.7
DD17b-L7	/Cy5/GA+G CGG ATG GCG TAG +GCA<br>CTC GCC TAC CGC ATC CGT	—	—	—	—	65.2	65.4
DD17b-L7”	/Cy5/GA+G CGG ATG GCG TAG +GCA<br>ACACC CTC GCC TAC CGC ATC CGT ATCAA	—	—	—	—	66.3	66.2
DD17c-L1	/ATTO532/GAG CGG AT+G +G+CG TAG GCA<br>CTC GCC TAC CGC ATC CGT	—	—	—	—	66.2	67.6
DD17c-L1”	/ATTO532/GAG CGG AT+G +G+CG TAG GCA<br>ACACC CTC GCC TAC CGC ATC CGT ATCAA	—	—	—	—	67.3	65.5
DD17c-L2	/ATTO532/GAG CGG AT+G GCG TAG GCA<br>CTC GCC TAC CGC ATC CGT	—	—	—	—	61.4	62.5
DD17c-L2”	/ATTO532/GAG CGG AT+G GCG TAG GCA<br>ACACC CTC GCC TAC CGC ATC CGT ATCAA	—	—	—	—	63.6	63.9
DD17c-L3	/ATTO532/GAG CGG ATG +GCG TAG GCA<br>CTC GCC TAC CGC ATC CGT	—	—	—	—	65.4	64.4
DD17c-L3”	/ATTO532/GAG CGG ATG +GCG TAG GCA<br>ACACC CTC GCC TAC CGC ATC CGT ATCAA	—	—	—	—	66.1	64.3
DD17c-L4	/ATTO532/GAG CGG ATG G+CG TAG GCA<br>CTC GCC TAC CGC ATC CGT	—	—	—	—	65.2	65.3
DD17c-L4”	/ATTO532/GAG CGG ATG G+CG TAG GCA<br>ACACC CTC GCC TAC CGC ATC CGT ATCAA	—	—	—	—	67.0	65.3
DD17c-L5	/ATTO532/GAG CGG AT+G +GCG TAG GCA<br>CTC GCC TAC CGC ATC CGT	—	—	—	—	65.2	65.3

(continued on next page)

(5 continued)

identification	Duplex	1.0 μM T_{exp}	1.0 μM T_{pre}	0.6 μM T_{exp}	0.6 μM T_{pre}	0.3 μM T_{exp}	0.3 μM T_{pre}
DD17c-L5”	/ATTO532/GAG CGG AT+G +GCG TAG GCA<br>ACACC CTC GCC TAC CGC ATC CGT ATCAA	—	—	—	—	67.3	65.3
DD17c-L6	/ATTO532/GA+G CGG AT+G GCG TAG GCA<br>CTC GCC TAC CGC ATC CGT	—	—	—	—	61.5	62.3
DD17c-L6”	/ATTO532/GA+G CGG AT+G GCG TAG GCA<br>ACACC CTC GCC TAC CGC ATC CGT ATCAA	—	—	—	—	63.2	62.8
DD17c-L7	/ATTO532/GA+G CGG ATG GCG TAG +GCA<br>CTC GCC TAC CGC ATC CGT	—	—	—	—	62.1	65.2
DD17c-L7”	/ATTO532/GA+G CGG ATG GCG TAG +GCA<br>ACACC CTC GCC TAC CGC ATC CGT ATCAA	—	—	—	—	63.5	65.2
DD17d-L1	/ATTO647/GAG CGG AT+G +G+CG TAG GCA<br>CTC GCC TAC CGC ATC CGT	—	—	—	—	74.6	72.9
DD17d-L1”	/ATTO647/GAG CGG AT+G +G+CG TAG GCA<br>ACACC CTC GCC TAC CGC ATC CGT ATCAA	—	—	—	—	78.2	73.0
DD17d-L2	/ATTO647/GAG CGG AT+G GCG TAG GCA<br>CTC GCC TAC CGC ATC CGT	—	—	—	—	68.5	69.5
DD17d-L2”	/ATTO647/GAG CGG AT+G GCG TAG GCA<br>ACACC CTC GCC TAC CGC ATC CGT ATCAA	—	—	—	—	71.1	68.9
DD17d-L3	/ATTO647/GAG CGG ATG +GCG TAG GCA<br>CTC GCC TAC CGC ATC CGT	—	—	—	—	69.3	72.6
DD17d-L3”	/ATTO647/GAG CGG ATG +GCG TAG GCA<br>ACACC CTC GCC TAC CGC ATC CGT ATCAA	—	—	—	—	71.2	71.8
DD17d-L4	/ATTO647/GAG CGG ATG G+CG TAG GCA<br>CTC GCC TAC CGC ATC CGT	—	—	—	—	73.1	72.9
DD17d-L4”	/ATTO647/GAG CGG ATG G+CG TAG GCA<br>ACACC CTC GCC TAC CGC ATC CGT ATCAA	—	—	—	—	74.4	74.1
DD17d-L5	/ATTO647/GAG CGG AT+G +GCG TAG GCA<br>CTC GCC TAC CGC ATC CGT	—	—	—	—	72.0	74.1
DD17d-L5”	/ATTO647/GAG CGG AT+G +GCG TAG GCA<br>ACACC CTC GCC TAC CGC ATC CGT ATCAA	—	—	—	—	73.8	73.3
DD17d-L6	/ATTO647/GA+G CGG AT+G GCG TAG GCA<br>CTC GCC TAC CGC ATC CGT	—	—	—	—	68.7	69.9
DD17d-L6”	/ATTO647/GA+G CGG AT+G GCG TAG GCA<br>ACACC CTC GCC TAC CGC ATC CGT ATCAA	—	—	—	—	70.3	69.6
DD17d-L7	/ATTO647/GA+G CGG ATG GCG TAG +GCA<br>CTC GCC TAC CGC ATC CGT	—	—	—	—	69.4	71.1
DD17d-L7”	/ATTO647/GA+G CGG ATG GCG TAG +GCA<br>ACACC CTC GCC TAC CGC ATC CGT ATCAA	—	—	—	—	71.0	71.9

Supplementary Table 6: Notation for overhangs either at the 3′ or 5′. Code V (void) stands for the unmatched opposite strand.

3′ overhang	code	overhang 5′
ACACCATCAA	L	
AGAAAGGAGA	F	
AGGTGAGGTA	S	
AGGTG	Q	
ACACC	P	
AGAAA	H	
	D	GGAGA
	M	ATCAA
	E	AGGTA
void	V	void

Supplementary Table 7: Notation for fluorescent markers.

marker	code	marker	code
Cy3	B	Cy5	R
ATTO532	J	ATTO647	I

Supplementary Table 8: Morse potentials D in meV, calculated in Step 2 for subset O (duplexes with and without overhangs, but no fluorophores). BP^{XV} stands for base pairs (BP) next to an overhang of type XV. For instance, CG^{PV} is a CG base pair next to the overhang ACACC (P).

Base-pair	D	overhang	D	Base-pair	D	overhang	D
AT	37.9 ± 5.5			CG	64.9 ± 6.9		
AT^{DV}	20.0 ± 6.5	DV	15.4 ± 0.83	CG^{FV}	24.7 ± 4.7	FV	7.18 ± 0.80
AT^{EV}	8.02 ± 1.3	EV	13.2 ± 0.092	CG^{HV}	22.5 ± 6.2	HV	17.3 ± 1.2
AT^{MV}	26.0 ± 14	MV	10.1 ± 1.3	CG^{LV}	38.3 ± 4.5	LV	14.1 ± 0.80
AT^{QV}	4.41 ± 0.31	QV	12.6 ± 0.064	CG^{PV}	38.8 ± 7.5	PV	4.99 ± 0.17
AT^{SV}	6.39 ± 0.77	SV	12.2 ± 0.63				

Supplementary Table 9: Stacking potentials k in eV/nm^2, calculated in Step 2 for subset O (duplexes with and without overhangs, but no fluorophores).

NN	k	NN	k
AT-AT (AA/TT)	2.79 ± 0.37	AT-CG (AC/TG)	2.69 ± 0.37
AT-GC (AG/TC)	4.56 ± 0.78	AT-TA (AT/TA)	1.92 ± 0.17
CG-AT (CA/GT)	2.29 ± 0.51	CG-CG (CC/GG)	2.26 ± 0.61
CG-GC (CG/GC)	2.71 ± 0.50	GC-AT (GA/CT)	2.89 ± 0.39
GC-CG (GC/CG)	3.12 ± 0.74	TA-AT (TA/AT)	2.36 ± 0.48

Supplementary Table 10: Morse potentials D in meV, calculated in Step 3, for set F containing the fluorophores. BP^X stands for base pairs (BP) next to a fluorescent marker X, for instance AT^B is a AT base pair next to a Cy3 (B) fluorophore.

Base-pair	D	Base-pair	D	free marker	D
AT^B	95.0 ± 5.4	CG^B	12.4 ± 6.0	BV	32.9 ± 4.3
AT^I	81.2 ± 27	CG^I	100 ± 13	IV	54.4 ± 18
AT^J	22.8 ± 6.7	CG^J	114 ± 27	JV	20.9 ± 8.1
AT^R	37.7 ± 1.4	CG^R	73.5 ± 13	RV	33.4 ± 5.2

Supplementary Table 11: Morse potentials D in meV for florescent markers with overhangs, calculated in Step 3 (set F containing the fluorophores).

overhang (code)	Cy3	Cy5	PEP	PER
AGAAAGGAGA(F)	92.6 ± 13	38.4 ± 2.6	31.8 ± 8.9	37.4 ± 9.9
AGAAA(H)	89.5 ± 19	50.3 ± 37	36.3 ± 4.5	46.6 ± 14
ACACCATCAA(L)	28.5 ± 8.4	36.7 ± 4.6	26.5 ± 8.8	29.6 ± 6.5
ACACC(P)	273 ± 33	38.5 ± 3.4	27.7 ± 11	22.2 ± 7.3
AGGTG(Q)	28.9 ± 5.2	38.1 ± 2.0	121 ± 8.1	71.6 ± 28
AGGTGAGGTA(S)	42.0 ± 6.9	36.9 ± 2.4	41.8 ± 8.9	54.4 ± 18

Supplementary Table 12: Stacking potentials k, in eV/nm^2, of base-pairs next to fluorescent markers, calculated in Step 3 (set F containing the fluorophores).

NN	k	NN	k	NN	k	NN	k
AT^B-GC	2.59 ± 0.22	AT^I-GC	1.72 ± 0.68	AT^J-GC	2.42 ± 0.13	AT^R-GC	2.46 ± 0.13
BF-GC^B	2.49 ± 0.046	BH-GC^B	2.53 ± 0.092	BL-GC^B	2.18 ± 0.42	BP-GC^B	2.02 ± 1.2
BQ-AT^B	2.41 ± 0.22	BS-AT^B	2.46 ± 0.13	BV-AT^B	2.50 ± 0.091	BV-GC^B	2.31 ± 0.24
CG^I-FI	2.55 ± 0.28	CG^I-HI	2.48 ± 0.54	CG^I-LI	2.19 ± 0.79	CG^I-PI	1.73 ± 0.70
CG^I-VI	1.00 ± 0.67	CG^J-FJ	2.56 ± 0.25	CG^J-HJ	2.41 ± 0.27	CG^J-LJ	2.21 ± 0.51
CG^J-PJ	1.69 ± 0.73	CG^J-VJ	2.54 ± 0.60	CG^R-FR	2.50 ± 0.015	CG^R-HR	2.51 ± 0.13
CG^R-LR	2.44 ± 0.21	CG^R-PR	2.47 ± 0.080	CG^R-VR	2.26 ± 0.48	GC^B-AT	1.96 ± 0.61
GC^I-AT	1.98 ± 0.58	GC^J-AT	2.16 ± 1.3	GC^R-AT	2.48 ± 0.11	IQ-AT^I	3.35 ± 3.2
IS-AT^I	0.977 ± 0.62	IV-AT^I	4.24 ± 2.4	JQ-AT^J	2.92 ± 0.59	JS-AT^J	2.49 ± 0.13
JV-AT^J	2.45 ± 0.15	RQ-AT^R	2.50 ± 0.030	RS-AT^R	2.51 ± 0.044	RV-AT^R	2.50 ± 0.050

Supplementary Table 13: Morse potentials D for LNA modified bases in meV, calculated in Step 4 (set L, only duplexes containing LNA modifications).

base pair	D	base pair	D
C+G	65.0 ± 10	G+C	99.0 ± 36

Supplementary Table 14: Stacking potentials k for LNA modified bases in eV/nm^2, calculated in Step 4 (set L, only duplexes containing LNA modifications).

NN	k	NN	k	NN	k
AT-+GC (A+G/TC)	1.95 ± 1.8	CG-C+G (CC/G+G)	0.00460 ± 0.0084	CG-G+C (CG/G+C)	2.61 ± 1.7
C+G-AT (CA/+GT)	4.32 ± 2.4	C+G-CG (CC/+GG)	1.34 ± 1.8	C+G-C+G (CC/+G+G)	3.66 ± 1.5
GC-C+G (GC/C+G)	3.65 ± 3.0	GC-+CG (G+C/CG)	2.68 ± 1.1	G+C-C+G (GC/+C+G)	3.36 ± 1.8

Supplementary Note 2. DNA/LNA:RNA (DRL) optimization

The DNA/LNA:RNA (DRL) sequences contained a mix of LNA and DNA/RNA mismatches, shown in supplementary table 15. Due to the large number of variables we could not calculate the canonical DNA/RNA parameters as we had for DNA/LNA:DNA. Instead, we prepared a new set for low-salt concentration from an independent sequence set (DRLS, see below for notation) that we recently used for calculation of nearest-neighbours parameters.[12]

2.1 Data set description

Set DRLS DNA/RNA melting temperatures at low-salt concentrations from Ref. 12

Set DLR DNA/RNA sequences some of which containing LNA modification, Cy3/Cy5 fluorophores, and mismatches, in the absence of crowding agents, shown in supplementary table 15.

2.2 Parametrization workflow

Step 1 Parametrization of set DRLS following the procedure outline in Ref. 13. The resulting Morse potentials are shown in Table 16 and stacking parameters in Table 17.

Step 2 Parametrization of set DLR using the parameters of Step 1 as input. Only Morse potentials were calculated as there were not enough sequences to also include additional 33 stacking parameters. The calculated Morse potentials are shown in Table. 18.

Supplementary Table 15: Sequences for the DLR set of duplexes. Mismatches are shown in grey shaded boxes, and LNA are shown in red and with a plus in from of corresponding nucleotide letter. Fluorophores are shown in green. DNA strands are shown in 5′ →3′ on top of RNA strands in 3′ ←5′ direction. The final quality parameters of the optimization were $\chi^2 = 590$ °C^2 and $\langle \Delta T \rangle = 2.4$ °C.

identification	Duplex	0.125 μM T_{exp}	0.125 μM T_{pre}	0.5 μM T_{exp}	0.5 μM T_{pre}
DR1	/Cy3/d(TAG CTA CAG AGA AAT CTC GAT)<br>r(AUC GAU GUC UCU UUA GAG CUA)/Cy5/	57.5	52.8	58.5	54.8
DR1-CTRL	d(TAG CTA CAG AGA AAT CTC GAT)<br>r(AUC GAU GUC UCU UUA GAG CUA)	54.0	53.1	57.0	55.1
DR2	/Cy3/d(TAG CTA CAG A GA AAT CTC GAT)<br>r(AUC GAU GUC A CU UUA GAG CUA)/Cy5/	52.0	51.4	55.0	53.5
DR2-CTRL	d(TAG CTA CAG A GA AAT CTC GAT)<br>r(AUC GAU GUC A CU UUA GAG CUA)	54.0	51.6	57.0	53.7
DR3	/Cy3/d(ACT GTA CAT GAG AAA CTT TTT CTC)<br>r(UGA CAU GUA CUC UUU GAA AAA GAG)/Cy5/	57.0	60.3	58.0	62.1
DR3-CTRL	d(ACT GTA CAT GAG AAA CTT TTT CTC)<br>r(UGA CAU GUA CUC UUU GAA AAA GAG)	55.0	60.7	56.0	62.6
DR4-CTRL	d(A CT GTA CAT GAG AAA CTT TTT CTC)<br>r(C GA CAU GUA CUC UUU GAA AAA GAG)	52.5	60.0	54.0	61.9
DR4	/Cy3/d(A CT GTA CAT GAG AAA CTT TTT CTC)<br>r(C GA CAU GUA CUC UUU GAA AAA GAG)/Cy5/	56.0	59.5	57.5	61.4
DR5L-CTRL	d(GTT GGA GCT +G +A +T GGC GTA GGC)<br>r(CAA CCU CGA C U A CCG CAU CCG)	62.0	65.3	66.0	67.0
DR5L	/Cy3/d(GTT GGA GCT +G +A +T GGC GTA GGC)<br>r(CAA CCU CGA C U A CCG CAU CCG)/Cy5/	67.0	65.3	69.5	67.0
DR6L-CTRL	d(GTT GGA GCT +G +A +T GGC GTA GGC)<br>r(CAA CCU CGA C C A CCG CAU CCG)	59.5	63.3	62.0	65.0

(continued on next page)

(5 continued)

identification	Duplex	0.125 μM T_{exp}	0.125 μM T_{pre}	0.5 μM T_{exp}	0.5 μM T_{pre}
DR6L	/Cy3/d(GTT GGA GCT +G +A +T GGC GTA GGC)<br>r(CAA CCU CGA C C A CCG CAU CCG)/Cy5/	61.2	63.3	64.0	65.1
DR9-CTRL	d(CTC CTG GGC TCA AGC AAT TCT)<br>r(GAG GAC CCG AGU UCG UUA AGA)	64.0	61.2	65.0	63.1
DR9	/Cy3/d(CTC CTG GGC TCA AGC AAT TCT)<br>r(GAG GAC CCG AGU UCG UUA AGA)/Cy5/	61.0	61.7	63.5	63.6
DR10	/Cy3/d(CTC CTG GG C TCA AGC AAT TCT)<br>r(GAG GAC CC U AGU UCG UUA AGA)/Cy5/	60.0	61.2	61.0	63.0
DR10-CTRL	d(CTC CTG GG C TCA AGC AAT TCT)<br>r(GAG GAC CC U AGU UCG UUA AGA)	62.5	60.7	64.0	62.6
DR11-CTRL	d(CAG CCT CCC ACG TAG CTG GGA)<br>r(GUC GGA GGG UGC AUC GAC CCU)	70.0	64.6	71.0	66.3
DR11	/Cy3/d(CAG CCT CCC ACG TAG CTG GGA)<br>r(GUC GGA GGG UGC AUC GAC CCU)/Cy5/	67.0	65.0	69.5	66.7
DR12-CTRL	d(CAG CCT CCC A C G TAG CTG GGA)<br>r(GUC GGA GGG U C C AUC GAC CCU)	68.5	65.7	69.5	67.4
DR12	/Cy3/d(CAG CCT CCC A C G TAG CTG GGA)<br>r(GUC GGA GGG U C C AUC GAC CCU)/Cy5/	65.5	66.1	67.0	67.8
DR13L	/Cy3/d(AGT AGA GAC +G +C +G GTT TCA CCA)<br>r(UCA UCU CUG C G C CAA AGU GGU)/Cy5/	70.0	66.0	71.5	67.8
DR13-CTRL	d(AGT AGA GAC GCG GTT TCA CCA)<br>r(UCA UCU CUG CGC CAA AGU GGU)	64.0	61.6	65.5	63.4
DR13	/Cy3/d(AGT AGA GAC GCG GTT TCA CCA)<br>r(UCA UCU CUG CGC CAA AGU GGU)/Cy5/	65.0	61.5	66.5	63.4
DR13L-CTRL	d(AGT AGA GAC +G +C +G GTT TCA CCA)<br>r(UCA UCU CUG C G C CAA AGU GGU)	68.0	65.9	70.5	67.7
DR14	/Cy3/d(AGT AGA GAC G C G GTT TCA CCA)<br>r(UCA UCU CUG C C C CAA AGU GGU)/Cy5/	63.5	63.4	64.0	65.2
DR14-CTRL	d(AGT AGA GAC G C G GTT TCA CCA)<br>r(UCA UCU CUG C C C CAA AGU GGU)	62.5	63.3	64.0	65.1
DR14L	/Cy3/d(AGT AGA GAC +G +C +G GTT TCA CCA)<br>r(UCA UCU CUG C C C CAA AGU GGU)/Cy5/	66.0	65.2	68.5	66.9
DR14L-CTRL	d(AGT AGA GAC +G +C +G GTT TCA CCA)<br>r(UCA UCU CUG C C C CAA AGU GGU)	64.0	65.1	66.5	66.9
DR15L	/Cy3/d(GTT AGG TTG +G +T +C TCA AAC TCC)<br>r(CAA UCC AAC C A G AGU UUG AGG)/Cy5/	66.0	63.4	68.0	65.1
DR15-CTRL	d(GTT AGG TTG GTC TCA AAC TCC)<br>r(CAA UCC AAC CAG AGU UUG AGG)	59.0	60.1	61.0	62.0
DR15L-CTRL	d(GTT AGG TTG +G +T +C TCA AAC TCC)<br>r(CAA UCC AAC C A G AGU UUG AGG)	65.0	63.4	66.0	65.2
DR15	/Cy3/d(GTT AGG TTG GTC TCA AAC TCC)<br>r(CAA UCC AAC CAG AGU UUG AGG)/Cy5/	59.0	59.9	61.0	61.8
DR16	/Cy3/d(GTT AGG TTG G T C TCA AAC TCC)<br>r(CAA UCC AAC C C G AGU UUG AGG)/Cy5/	58.0	58.9	59.0	60.8
DR16-CTRL	d(GTT AGG TTG G T C TCA AAC TCC)<br>r(CAA UCC AAC C C G AGU UUG AGG)	57.5	59.3	59.5	61.1
DR16L-CTRL	d(GTT AGG TTG +G +T +C TCA AAC TCC)<br>r(CAA UCC AAC C C G AGU UUG AGG)	60.5	63.2	62.0	65.0
DR16L	/Cy3/d(GTT AGG TTG +G +T +C TCA AAC TCC)<br>r(CAA UCC AAC C C G AGU UUG AGG)/Cy5/	62.0	63.1	63.5	64.9

Supplementary Table 16: Morse potentials D for DNA/RNA in meV calculated in Step 1, section 2.2.

Base-pair	D	Base-pair	D
dArU	12.4 ± 9.7	dTrA	38.7 ± 8.9
dCrG	79.2 ± 16	dGrC	60.8 ± 13

Supplementary Table 17: Stacking potentials k for DNA/RNA in eV/nm^2, calculated in Step 1, section 2.2.

NN	k	NN	k	NN	k	NN	k
dArU-dArU	0.357 ± 0.63	dArU-dCrG	3.63 ± 1.7	dArU-dGrC	6.26 ± 2.5	dArU-dTrA	0.394 ± 0.68
dCrG-dArU	5.94 ± 3.1	dCrG-dCrG	2.32 ± 1.2	dCrG-dGrC	1.10 ± 0.92	dCrG-dTrA	4.56 ± 2.3
dGrC-dArU	4.07 ± 1.5	dGrC-dCrG	1.53 ± 0.68	dTrA-dCrG	1.86 ± 0.64	dGrC-dTrA	1.70 ± 0.98
dTrA-dArU	1.51 ± 1.4	dGrC-dGrC	1.53 ± 0.58	dTrA-dGrC	6.11 ± 2.9	dTrA-dTrA	3.10 ± 1.3

Supplementary Table 18: Morse potentials D for DNA/LNA:RNA in meV calculated in Step 2, section 2.2.

Base-pair	D	Base-pair	D	Base-pair	D
dArA	8.97 ± 21	dArC	0.00900 ± 0.013	dCrC	167 ± 24
dCrU	58.5 ± 5.4	dTrC	0.0190 ± 0.023		
d+CrG	176 ± 43	d+ArC	0.0100 ± 0.012	d+GrC	211 ± 49
d+ArU	137 ± 34	d+TrC	0.0250 ± 0.049	d+TrA	10.9 ± 11
dCy3rCy5	1.27 ± 2.9				

Supplementary References

[1] L. Miotke, M. Barducci, K. Astakhova, Novel signal-enhancing approaches for optical detection of nucleic acids—going beyond target amplification, Chemosensors 3 (3) (2015) 224–240.

[2] G. Weber, J. W. Essex, C. Neylon, Probing the microscopic flexibility of DNA from melting temperatures, Nat. Phys. 5 (2009) 769–773. doi:10.1038/nphys1371.

[3] G. Weber, Mesoscopic model parametrization of hydrogen bonds and stacking interactions of RNA from melting temperatures, Nucleic Acids Res. 41 (2013) e30. doi:10.1093/nar/gks964. URL http://nar.oxfordjournals.org/content/41/1/e30

[4] M. Peyrard, A. R. Bishop, Statistical mechanics of a nonlinear model for DNA denaturation, Phys. Rev. Lett. 62 (23) (1989) 2755–2757.

[5] P. M. Morse, Diatomic molecules according to the wave mechanics. II. Vibrational levels, Physical Review 34 (1) (1929) 57.

[6] G. Weber, N. Haslam, J. W. Essex, C. Neylon, Thermal equivalence of DNA duplexes for probe design, J. Phys.: Condens. Matter 21 (2009) 034106. doi:10.1088/0953-8984/21/3/034106.

[7] G. Weber, N. Haslam, N. Whiteford, A. Prügel-Bennett, J. W. Essex, C. Neylon, Thermal equivalence of DNA duplexes without melting temperature calculation, Nat. Phys. 2 (2006) 55–59. doi:10.1038/nphys189.

[8] W. H. Press, S. A. Teukolsky, W. T. Vetterling, B. P. Flannery, Numerical Recipes in C, Cambridge University Press, Cambridge, 1988.

[9] G. Weber, TfReg: Calculating DNA and RNA melting temperatures and opening profiles with mesoscopic models, Bioinformatics 29 (2013) 1345–1347. doi:10.1093/bioinformatics/btt133.
URL http://bioinformatics.oxfordjournals.org/content/29/10/1345

[10] Y.-L. Zhang, W.-M. Zheng, J.-X. Liu, Y. Z. Chen, Theory of DNA melting based on the Peyrard-Bishop model, Phys. Rev. E 56 (6) (1997) 7100–7115.

[11] P. Miranda, L. M. Oliveira, G. Weber, Mesoscopic modelling of Cy3 and Cy5 dyes attached to DNA duplexes, Biophys. Chem. 230C (2017) 62–67. doi:10.1016/j.bpc.2017.08.007.
URL http://www.sciencedirect.com/science/article/pii/S0301462217302831

[12] V. B. Barbosa, E. de Oliveira Martins, G. Weber, Nearest-neighbour parameters optimized for melting temperature prediction of DNA/RNA hybrids at high and low salt concentrations, Biophys. Chem. 251C (2019) 106189. doi:10.1016/j.bpc.2019.106189.

[13] E. d. O. Martins, V. B. Barbosa, G. Weber, DNA/RNA hybrid mesoscopic model shows strong stability dependence with deoxypyrimidine content and stacking interactions similar to RNA/RNA, Chem. Phys. Lett. 715C (2019) 14–19. doi:10.1016/j.cplett.2018.11.015.

[14] Cao J, Zhu B, Zheng K, et al. Recent Progress in NIR-II Contrast Agent for Biological Imaging. *Front Bioeng Biotechnol*. 2020;7:487. doi:10.3389/fbioe.2019.00487

[15] Babion I, Snoek BC, van de Wiel MA, Wilting SM, Steenbergen RDM. A Strategy to Find Suitable Reference Genes for miRNA Quantitative PCR Analysis and Its Application to Cervical Specimens. *J Mol Diagn*. 2017;19(5):625-637. doi:10.1016/j.jmoldx.2017.04.010

Supplementary note 3. Bead-bait hybridization assay.

Procedures and results

For *BRAF* and *EGFR* control study, we used bait probe designs given in Table 1 of the main paper.

For *KRAS* G12D detection, we designed a range of bait probes specific to the mutation. In the probe design, our main goal has been to create a least stable probe:WT RNA complex, vs. a stable binding of probe:mutant (full match) RNA.

Computing (described in section 5, Supplementary information) suggested the following strategies for probe design:

- avoid having +G and +G+G in the probe;[1]
- consider not sequential incorporation of LNAs in the area of a mismatch;
- include dTrC and dCrU mismatches;
- avoid dArU base pair stretches.

Two other approaches we tested for the probe design were adding an additional mismatch to the probe, to destabilize the probe:wild type complex event further, and moving a probe to periphery/terminal region along the target, thus promoting more active opening in case of a mismatched nucleotide.

The resulting probe designs are given in Supplementary Table 19, below.

[1] LNA is labelled as a plus in front of corresponding nucleotide letter.

Supplementary Table 19. Design strategy for *KRAS* G12D detection in *KRAS* G12D RNA.

Duplex code	Mismatch(es)	Duplex probe:target RNA sequences	Target RNA name
DR5-CTRL,DR5L-CTRL	-	5’- GTT GGA GCT +G+A+T GGC GTA GGC<br>3’- rCrArA rCrCrU rCrGrA rC**r**UrA rCrCrG rCrArU rCrCrG	*KRAS G12D*
DR6-CTRL, DR6L-CTRL	dArC<br>+ArC	5’- GTT GGA GCT +G+A+T GGC GTA GGC<br>3’- rCrArA rCrCrU rCrGrA rC**r**CrA rCrCrG rCrArU rCrCrG	*KRAS wt*
DR5L1-CTRL	-	5’- GTT GGA G+CT G+AT GG+C GTA GGC<br>3’-rCrArA rCrCrU rCrGrA rCrUrA rCrCrG rCrArU rCrCrG	*KRAS G12D*
DR6L1-CTRL	dArC<br>+ArC	5’- GTT GGA G+CT G+AT GG+C GTA GGC<br>3’-rCrArA rCrCrU rCrGrA rCrCrA rCrCrG rCrArU rCrCrG	*KRAS wt*
DR5L2-CTRL	-	5’- GTT GGA G+CT G+AT GGC GTA GGC<br>3’-rCrArA rCrCrU rCrGrA rCrUrA rCrCrG rCrArU rCrCrG	*KRAS G12D*
DR5L2-CTRL	dArC<br>+ArC	5’- GTT GGA G+CT G+AT GGC GTA GGC<br>3’-rCrArA rCrCrU rCrGrA rCrCrA rCrCrG rCrArU rCrCrG	*KRAS wt*
DR17-CTRL, DR17L1-CTRL	dTrC	5’- GTT GGA G+CT ***T******+AT GGC GTA GGC<br>3’-rCrArA rCrCrU rCrGrA rCrUrA rCrCrG rCrArU rCrCrG	*KRAS G12D*
DR18-CTRL, DR18L1-CTRL	dArC,dTrC<br>+ArC, dTrC	5’- GTT GGA G+CT ***T******+AT GGC GTA GGC<br>3’-rCrArA rCrCrU rCrGrA rCrCrA rCrCrG rCrArU rCrCrG	*KRAS wt*
DR17L2-CTRL	dTrC	5’- GTT GGA G+CT ***T******+AT GG+C GTA GGC<br>3’-rCrArA rCrCrU rCrGrA rCrUrA rCrCrG rCrArU rCrCrG	*KRAS G12D*
DR18L2-CTRL	dArC,dTrC<br>+ArC, dTrC	5’- GTT GGA G+CT ***T******+AT GG+C GTA GGC<br>3’-rCrArA rCrCrU rCrGrA rCrCrA rCrCrG rCrArU rCrCrG	*KRAS wt*
DR17L3-CTRL	dTrC	5’- GTT GGA GCT ***T******+AT GG+C GTA GGC<br>3’-rCrArA rCrCrU rCrGrA rCrUrA rCrCrG rCrArU rCrCrG	*KRAS G12D*
DR18L3-CTRL	dArC,dTrC<br>+ArC, dTrC	5’- GTT GGA GCT ***T******+AT GG+C GTA GGC<br>3’-rCrArA rCrCrU rCrGrA rCrCrA rCrCrG rCrArU rCrCrG	*KRAS wt*
DR19-CTRL, DR19L1-CTRL	-	5’- G+AT GG+C G+TA GGC AAG AGT GC<br>3’-rCrUrA rCrCrG rCrArU rCrCrG rUrUrC rUrCrA rCrG	*KRAS G12D*
DR20-CTRL, DR20L1-CTRL	dArC<br>+ArC	5’- G+AT GG+C G+TA GGC AAG AGT GC<br>3’-rCrCrA rCrCrG rCrArU rCrCrG rUrUrC rUrCrA rCrG	*KRAS wt*
DR21-CTRL, DR21L1-CTRL	dTrC	5’- ***T******+AT GG+C G+TA GGC AAG AGT GC<br>3’-rCrUrA rCrCrG rCrArU rCrCrG rUrUrC rUrCrA rCrG	*KRAS G12D*
DR22-CTRL, DR22L1-CTRL	dArC,dTrC<br>+ArC, dTrC	5’- ***T******+AT GG+C G+TA GGC AAG AGT GC<br>3’-rCrCrA rCrCrG rCrArU rCrCrG rUrUrC rUrCrA rCrG	*KRAS wt*
DR23-CTRL, DR23L1-CTRL	dTrC	5’- G+AT ***T******G+C G+TA GGC AAG AGT GC<br>3’-rCrUrA rCrCrG rCrArU rCrCrG rUrUrC rUrCrA rCrG	*KRAS G12D*
DR24-CTRL, DR24L1-CTRL	dArC,dTrC<br>+ArC, dTrC	5’- G+AT ***T******G+C G+TA GGC AAG AGT GC<br>3’-rCrCrA rCrCrG rCrArU rCrCrG rUrUrC rUrCrA rCrG	*KRAS wt*
DR25-CTRL, DR25L1-CTRL	-	5’- +AT GG+C G+TA GGC AAG AGT GCC<br>3’-rUrA rCrCrG rCrArU rCrCrG rUrUrC rUrCrA rCrGrG	*KRAS G12D*
DR26-CTRL, DR26L1-CTRL	dArC<br>+ArC	5’- +AT GG+C G+TA GGC AAG AGT GCC<br>3’-rCrA rCrCrG rCrArU rCrCrG rUrUrC rUrCrA rCrGrG	*KRAS wt*
DR27-CTRL, DR27L1-CTRL	dTrC	5’- +AT ***T******G+C G+TA GGC AAG AGT GCC<br>3’- rUrA rCrCrG rCrArU rCrCrG rUrUrC rUrCrA rCrGrG	*KRAS G12D*

DR28-CTRL,<br>DR28L1-CTRL	dArC,dTrC<br>+ArC, dTrC	5'- +AT ***T****G+C G+TA GGC AAG AGT GCC<br>3'- **rC**rA rCrCrG rCrArU rCrCrG rUrUrC rUrCrA rCrGrG	*KRAS wt*
DR29-CTRL,<br>DR29L1-CTRL	dTrC	5'- +AT G***T*****+C G+TA GGC AAG AGT GCC<br>3'- **rU**rA rCrCrG rCrArU rCrCrG rUrUrC rUrCrA rCrGrG	*KRAS G12D*
DR30-CTRL,<br>DR30L1-CTRL	dArC,dTrC<br>+ArC, dTrC	5'- +AT G***T*****+C G+TA GGC AAG AGT GCC<br>3'- **rC**rA rCrCrG rCrArU rCrCrG rUrUrC rUrCrA rCrGrG	*KRAS wt*

Mismatch nucleotides are underlined; mismatch position in RNA is shown in bold. Additional mismatch is indicated in bold italized letter with a star. LNAs are shown in red and with a plus in front of corresponding nucleotide letter. Wt = wild-type.

All the duplexes given in Table 19 were then analyzed by computation, as described in Methods and Supplementary note 1. The results for predicted melting temperatures and difference (ΔT_m) for full match (mutant) vs mismatch (mutant probe:wild type RNA target), are shown in Supplementary Table 20, below.

Supplementary Table 20. Representative designs and theoretical prediction of T_m for probes with mutant (full match) and wild-type (mismatch) target.*

Probe sequence, 5'→3'	Target RNA sequence, 5'→3'	Probe name	T_m, °C	ΔT_m, °C
d(BGTTGGAGCTGATGGCGTAGGC)	r(RCAACCUCGACUACCGCAUCCG)	#DR5	62.30	
d(BGTTGGAGCTNWYGGCGTAGGC)	r(RCAACCUCGACUACCGCAUCCG)	#DR5L	65.30	
d(BGTTGGAGCTGATGGCGTAGGC)	r(RCAACCUCGACCACCGCAUCCG)	#DR6	62.50	-0.16
d(BGTTGGAGCTNWYGGCGTAGGC)	r(RCAACCUCGACCACCGCAUCCG)	#DR6L	63.30	1.95
d(GTTGGAGCTGATGGCGTAGGC)	r(CAACCUCGACUACCGCAUCCG)	#DR17-CTRL	62.30	
d(GTTGGAGZTGWTGGZGTAGGC)	r(CAACCUCGACUACCGCAUCCG)	#DR17L1-CTRL	67.10	
d(GTTGGAGCTGATGGCGTAGGC)	r(CAACCUCGACCACCGCAUCCG)	#DR18-CTRL	62.45	-0.13
d(GTTGGAGZTGWTGGZGTAGGC)	r(CAACCUCGACCACCGCAUCCG)	#DR18L1-CTRL	65.39	1.69
d(GTTGGAGZTGWTGGCGTAGGC)	r(CAACCUCGACUACCGCAUCCG)	#DR17L2-CTRL	65.54	
d(GTTGGAGZTGWTGGCGTAGGC)	r(CAACCUCGACCACCGCAUCCG)	#DR18L2-CTRL	63.71	1.82
d(GTTGGAGCTTATGGCGTAGGC)	r(CAACCUCGACUACCGCAUCCG)	#DR19-CTRL	60.11	
d(GTTGGAGZTTWTGGCGTAGGC)	r(CAACCUCGACUACCGCAUCCG)	#DR19L1-CTRL	64.44	
d(GTTGGAGCTTATGGCGTAGGC)	r(CAACCUCGACCACCGCAUCCG)	#DR20-CTRL	60.83	-0.72
d(GTTGGAGZTTWTGGCGTAGGC)	r(CAACCUCGACCACCGCAUCCG)	#DR20L1-CTRL	62.51	1.92
d(GTTGGAGZTTWTGGZGTAGGC)	r(CAACCUCGACUACCGCAUCCG)	#DR19L2-CTRL	65.98	
d(GTTGGAGZTTWTGGZGTAGGC)	r(CAACCUCGACCACCGCAUCCG)	#DR20L2-CTRL	64.38	1.59
d(GTTGGAGCTTWTGGZGTAGGC)	r(CAACCUCGACUACCGCAUCCG)	#DR19L3-CTRL	64.93	
d(GTTGGAGCTTWTGGZGTAGGC)	r(CAACCUCGACCACCGCAUCCG)	#DR20L3-CTRL	63.07	1.86
d(GATGGCGTAGGCAAGAGTGC)	r(CUACCGCAUCCGUUCUCACG)	#DR21-CTRL	58.76	
d(GWTGGZGYAGGCAAGAGTGC)	r(CUACCGCAUCCGUUCUCACG)	#DR21L1-CTRL	63.95	
d(GATGGCGTAGGCAAGAGTGC)	r(CCACCGCAUCCGUUCUCACG)	#DR22-CTRL	59.13	-0.36
d(GWTGGZGYAGGCAAGAGTGC)	r(CCACCGCAUCCGUUCUCACG)	#DR22L1-CTRL	61.55	2.40
d(TATGGCGTAGGCAAGAGTGC)	r(CUACCGCAUCCGUUCUCACG)	#DR23-CTRL	57.05	
d(TWTGGZGYAGGCAAGAGTGC)	r(CUACCGCAUCCGUUCUCACG)	#DR23L1-CTRL	62.87	
d(TATGGCGTAGGCAAGAGTGC)	r(CCACCGCAUCCGUUCUCACG)	#DR24-CTRL	57.28	-0.24
d(TWTGGZGYAGGCAAGAGTGC)	r(CCACCGCAUCCGUUCUCACG)	#DR24L1-CTRL	60.42	2.46
d(GATTGCGTAGGCAAGAGTGC)	r(CUACCGCAUCCGUUCUCACG)	#DR25-CTRL	55.58	
d(GWTTGZGYAGGCAAGAGTGC)	r(CUACCGCAUCCGUUCUCACG)	#DR25L1-CTRL	62.97	
d(GATTGCGTAGGCAAGAGTGC)	r(CCACCGCAUCCGUUCUCACG)	#DR26-CTRL	55.74	-0.16
d(GWTTGZGYAGGCAAGAGTGC)	r(CCACCGCAUCCGUUCUCACG)	#DR26L1-CTRL	59.87	3.10
d(ATGGCGTAGGCAAGAGTGCC)	r(UACCGCAUCCGUUCUCACGG)	#DR27-CTRL	59.48	
d(WTGGZGYAGGCAAGAGTGCC)	r(UACCGCAUCCGUUCUCACGG)	#DR27L1-CTRL	64.43	
d(ATGGCGTAGGCAAGAGTGCC)	r(CACCGCAUCCGUUCUCACGG)	#DR28-CTRL	59.88	-0.40
d(WTGGZGYAGGCAAGAGTGCC)	r(CACCGCAUCCGUUCUCACGG)	#DR28L1-CTRL	62.20	2.23
d(ATTGCGTAGGCAAGAGTGCC)	r(UACCGCAUCCGUUCUCACGG)	#DR29-CTRL	56.93	
d(WTTGZGYAGGCAAGAGTGCC)	r(UACCGCAUCCGUUCUCACGG)	#DR29L1-CTRL	63.43	
d(ATGGCGTAGGCAAGAGTGCC)	r(CACCGCAUCCGUUCUCACGG)	#DR30-CTRL	59.88	-2.95

d(WTTGZGYAGGCAAGAGTGCC)	r(CACCGCAUCCGUUCUCACGG)	#DR30L1-CTRL	60.66	2.76
d(ATGTCGTAGGCAAGAGTGCC)	r(UACCGCAUCCGUUCUCACGG)	#DR31-CTRL	57.77	
d(WTGTZGYAGGCAAGAGTGCC)	r(UACCGCAUCCGUUCUCACGG)	#DR31L1-CTRL	63.53	
d(ATGTCGTAGGCAAGAGTGCC)	r(CACCGCAUCCGUUCUCACGG)	#DR32-CTRL	58.11	-0.35
d(WTGTZGYAGGCAAGAGTGCC)	r(CACCGCAUCCGUUCUCACGG)	#DR32L1-CTRL	61.08	2.45

* ΔT_m is the difference for melting temperature of full match (mutant) vs mismatch (mutant probe:wild type RNA target).

The convertion table for DNA:LNA/RNA was: B {Cy3}, R {Cy5}, W {+A}; Y {+T}; N {+G}; Z {+C}, where plus indicates LNA.

Based on the highest mismatch discrimination potential, we selected ten probes from those given in Supplementary Tables 19 and 20. The probes, their fully matched and mismatched targets were purchased from Exiqon (LNA strands) and IDT (natural oligonucleotides).

We studied them by UV melting assay as described in Methods. In this case we used 125 nM concentration of oligonucleotides in 1X PBS, pH 7.2. The results along with a theoretical prediction for fully matched and mismatched DNA probe:RNA target duplexes are given in the Supplementary Table 21, below.

Supplementary Table 21. T_m results for selected designs – predicted and experimental values.

Duplex name (CTRL sequences)	Probe (5’→3’):target RNA (3’→5’)	Crowder	T_m match predicted,* °C	ΔT_m predicted, °C, match - mismatch	$\Delta\Delta T_m$,** °C Exper. – theor.
DR5-M1199	d({Cy3}GTTGGAGC{+T}G{+A}{+T}GGCGTAGGC)<br>r(CAA CCUCG U C U A CCGCAUCCG)	-	62.94	3.29	-0.56
DR5-M486	d({Cy3}GT{+T}GGAGC{+T}G{+A}TGGCGTAGGC)<br>r(CA A CCUCG U C U ACCGCAUCCG)	-	62.36	2.99	-1.16
DR5-M1331	d({Cy3}GTTGGAGCTG{+A}TGGCGTAGGC)<br>r(CAACCUCGUC U ACCGCAUCCG)	-	64.49	1.99	0.19
DR5-M140	d({Cy3}{+G}TTGGAGCT{+G}ATGGC{+G}TAGGC)<br>r(C AACCUCGU C UACCG C AUCCG)	-	67.61	-0.77	0.23
DR5-M1075	d({Cy3}GTTGGA{+G}CTGATGGC{+G}TA{+G}GC)<br>r(CAACCU C GUCUACCG C AU C CG)	-	66.87	-0.87	1.43
**DR6-M1199**	d({Cy3}GTTGGAGC{+T}G{+A}{+T}GGCGTAGGC)<br>r(CAA CCUCG U C C A CCGCAUCCG)	-	59.65		
**DR6-M486**	d({Cy3}GT{+T}GGAGC{+T}G{+A}TGGCGTAGGC)<br>r(CA A CCUCG U C C ACCGCAUCCG)	-	59.37		
**DR6-M1331**	d({Cy3}GTTGGAGCTG{+A}TGGCGTAGGC)<br>r(CAACCUCGUC C ACCGCAUCCG)	-	62.50		
**DR6-M140**	d({Cy3}{+G}TTGGAGCT{+G}ATGGC{+G}TAGGC)<br>r(C AACCUCGU C C ACCG C AUCCG)	-	68.38		
**DR6-M1075**	d({Cy3}GTTGGA{+G}CTGATGGC{+G}TA{+G}GC)<br>r(CAACCU C GUC C ACCG C AU C CG)	-	67.73		
DR5-M1199	d({Cy3}GTTGGAGC{+T}G{+A}{+T}GGCGTAGGC)<br>r(CAA CCUCG U C U A CCGCAUCCG)	DC	62.94	3.29	0.31
DR5-M486	d({Cy3}GT{+T}GGAGC{+T}G{+A}TGGCGTAGGC)<br>r(CA A CCUCG U C U ACCGCAUCCG)	DC	62.36	2.99	1.98
DR5-M1331	d({Cy3}GTTGGAGCTG{+A}TGGCGTAGGC)<br>r(CAACCUCGUC U ACCGCAUCCG)	DC	64.49	1.99	-1.29
DR5-M140	d({Cy3}{+G}TTGGAGCT{+G}ATGGC{+G}TAGGC)<br>r(C AACCUCGU C UACCG C AUCCG)	DC	67.61	-0.77	-1.30
DR5-M1075	d({Cy3}GTTGGA{+G}CTGATGGC{+G}TA{+G}GC)<br>r(CAACCU C GUCUACCG C AU C CG)	DC	66.87	-0.87	-0.81
**DR6-M1199**	d({Cy3}GTTGGAGC{+T}G{+A}{+T}GGCGTAGGC)<br>r(CAA CCUCG U C C A CCGCAUCCG)	DC	59.65		
**DR6-M486**	d({Cy3}GT{+T}GGAGC{+T}G{+A}TGGCGTAGGC)<br>r(CA A CCUCG U C C ACCGCAUCCG)	DC	59.37		
**DR6-M1331**	d({Cy3}GTTGGAGCTG{+A}TGGCGTAGGC)<br>r(CAACCUCGUC C ACCGCAUCCG)	DC	62.50		

**DR6-M140**	d({Cy3}{+G}TTGGAGCT{+G}ATGGC{+G}TAGGC)<br>r(C AACCUCGU C C ACCG C AUCCG)	DC	68.38		
**DR6-M1075**	d({Cy3}GTTGGA{+G}CTGATGGC{+G}TA{+G}GC)<br>r(CAACCU C GUC C ACCG C AU C CG)	DC	67.73		
DR5-M1199	d({Cy3}GTTGGAGC{+T}G{+A}{+T}GGCGTAGGC)<br>r(CAA CCUCG U C U A CCGCAUCCG)	mRNA	62.94	3.29	2.55
DR5-M486	d({Cy3}GT{+T}GGAGC{+T}G{+A}TGGCGTAGGC)<br>r(CA A CCUCG U C U ACCGCAUCCG)	mRNA	62.36	2.99	2.45
DR5-M1331	d({Cy3}GTTGGAGCTG{+A}TGGCGTAGGC)<br>r(CAACCUCGUC U ACCGCAUCCG)	mRNA	64.49	1.99	2.00
DR5-M140	d({Cy3}{+G}TTGGAGCT{+G}ATGGC{+G}TAGGC)<br>r(C AACCUCGU C UACCG C AUCCG)	mRNA	67.61	-0.77	-1.28
DR5-M1075	d({Cy3}GTTGGA{+G}CTGATGGC{+G}TA{+G}GC)<br>r(CAACCU C GUCUACCG C AU C CG)	mRNA	66.87	-0.87	-1.25
**DR6-M1199**	d({Cy3}GTTGGAGC{+T}G{+A}{+T}GGCGTAGGC)<br>r(CAA CCUCG U C C A CCGCAUCCG)	mRNA	59.65		
**DR6-M486**	d({Cy3}GT{+T}GGAGC{+T}G{+A}TGGCGTAGGC)<br>r(CA A CCUCG U C C ACCGCAUCCG)	mRNA	59.37		
**DR6-M1331**	d({Cy3}GTTGGAGCTG{+A}TGGCGTAGGC)<br>r(CAACCUCGUC C ACCGCAUCCG)	mRNA	62.50		
**DR6-M140**	d({Cy3}{+G}TTGGAGCT{+G}ATGGC{+G}TAGGC)<br>r(C AACCUCGU C C ACCG C AUCCG)	mRNA	68.38		
**DR6-M1075**	d({Cy3}GTTGGA{+G}CTGATGGC{+G}TA{+G}GC)<br>r(CAACCU C GUC C ACCG C AU C CG)	mRNA	67.73		
DR19-M1199	d(GTTGGAGC{+T}T{+A}{+T}GGCGTAGGC)<br>r(CAA CCUCG U C U A CCGCAUCCG)	-	61.74	4.52	-0.63
DR19-M510	d(GT{+T}GGAGCTT{+A}{+T}GGCGTAGGC)<br>r(CA A CCUCGUC U A CCGCAUCCG)	-	61.21	4.45	-2.30
DR19-M814	d(GTTG{+G}AGCTTA{+T}GG{+C}GTAGGC)<br>r(CAA C C UCGUCU A CC G CAUCCG)	-	64.27	0.09	-1.06
DR19-M492	d(GT{+T}GGAGC{+T}TATGGCG{+T}AGGC)<br>r(CA A CCUCG U CUACCGC A UCCG)	-	55.07	-1.27	-1.42
DR19-M227	d(G{+T}{+T}GGAGCTTATGGCG{+T}AGGC)<br>r(C A A CCUCGUCUACCGC A UCCG)	-	56.38	-1.34	-0.94
**DR20-M1199**	d(GTTGGAGC{+T}T{+A}{+T}GGCGTAGGC)<br>r(CAA CCUCG U C C A CCGCAUCCG)	-	57.23		
**DR20-M510**	d(GT{+T}GGAGCTT{+A}{+T}GGCGTAGGC)<br>r(CA A CCUCGUC C A CCGCAUCCG)	-	56.76		
**DR20-M814**	d(GTTG{+G}AGCTTA{+T}GG{+C}GTAGGC)<br>r(CAA C C UCGUCC A CC G CAUCCG)	-	64.18		
**DR20-M492**	d(GT{+T}GGAGC{+T}TATGGCG{+T}AGGC)<br>r(CA A CCUCG U C CACCGC A UCCG)	-	56.34		
**DR20-M227**	d(G{+T}{+T}GGAGCTTATGGCG{+T}AGGC)<br>r(C A A CCUCGUC C ACCGC A UCCG)	-	57.72		
DR19-M1199	d(GTTGGAGC{+T}T{+A}{+T}GGCGTAGGC)<br>r(CAA CCUCG U C U A CCGCAUCCG)	DC	61.74	4.52	3.68
DR19-M510	d(GT{+T}GGAGCTT{+A}{+T}GGCGTAGGC)<br>r(CA A CCUCGUC U A CCGCAUCCG)	DC	61.21	4.45	1.33

DR19-M814	d(GTTG{+G}AGCTTA{+T}GG{+C}GTAGGC)<br>r(CAA C C UCGUCU A CC G CAUCCG)	DC	64.27	0.09	-3.43
DR19-M492	d(GT{+T}GGAGC{+T}TATGGCG{+T}AGGC)<br>r(CA A CCUCG U CUACCGC A UCCG)	DC	55.07	-1.27	0.11
DR19-M227	d(G{+T}{+T}GGAGCTTATGGCG{+T}AGGC)<br>r(C A A CCUCGUCUACCGC A UCCG)	DC	56.38	-1.34	0.79
**DR20-M1199**	d(GTTGGAGC{+T}T{+A}{+T}GGCGTAGGC)<br>r(CAA CCUCG U C C A CCGCAUCCG)	DC	57.23		
**DR20-M510**	d(GT{+T}GGAGCTT{+A}{+T}GGCGTAGGC)<br>r(CA A CCUCGUC C A CCGCAUCCG)	DC	56.76		
**DR20-M814**	d(GTTG{+G}AGCTTA{+T}GG{+C}GTAGGC)<br>r(CAA C C UCGUCC A CC G CAUCCG)	DC	64.18		
**DR20-M492**	d(GT{+T}GGAGC{+T}TATGGCG{+T}AGGC)<br>r(CA A CCUCG U C CACCGC A UCCG)	DC	56.34		
**DR20-M227**	d(G{+T}{+T}GGAGCTTATGGCG{+T}AGGC)<br>r(C A A CCUCGUC C ACCGC A UCCG)	DC	57.72		
DR19-M1199	d(GTTGGAGC{+T}T{+A}{+T}GGCGTAGGC)<br>r(CAA CCUCG U C U A CCGCAUCCG)	mRNA	61.74	4.52	2.73
DR19-M510	d(GT{+T}GGAGCTT{+A}{+T}GGCGTAGGC)<br>r(CA A CCUCGUC U A CCGCAUCCG)	mRNA	61.21	4.45	3.85
DR19-M814	d(GTTG{+G}AGCTTA{+T}GG{+C}GTAGGC)<br>r(CAA C C UCGUCU A CC G CAUCCG)	mRNA	64.27	0.09	-1.59
DR19-M492	d(GT{+T}GGAGC{+T}TATGGCG{+T}AGGC)<br>r(CA A CCUCG U CUACCGC A UCCG)	mRNA	55.07	-1.27	-2.18
DR19-M227	d(G{+T}{+T}GGAGCTTATGGCG{+T}AGGC)<br>r(C A A CCUCGUCUACCGC A UCCG)	mRNA	56.38	-1.34	-1.93
**DR20-M1199**	d(GTTGGAGC{+T}T{+A}{+T}GGCGTAGGC)<br>r(CAA CCUCG U C C A CCGCAUCCG)	mRNA	57.23		
**DR20-M510**	d(GT{+T}GGAGCTT{+A}{+T}GGCGTAGGC)<br>r(CA A CCUCGUC C A CCGCAUCCG)	mRNA	56.76		
**DR20-M814**	d(GTTG{+G}AGCTTA{+T}GG{+C}GTAGGC)<br>r(CAA C C UCGUCC A CC G CAUCCG)	mRNA	64.18		
**DR20-M492**	d(GT{+T}GGAGC{+T}TATGGCG{+T}AGGC)<br>r(CA A CCUCG U C CACCGC A UCCG)	mRNA	56.34		
**DR20-M227**	d(G{+T}{+T}GGAGCTTATGGCG{+T}AGGC)<br>r(C A A CCUCGUC C ACCGC A UCCG)	mRNA	57.72		

Codes for mismatched duplexes are indicated in bold. In RNA sequences, mismatched nucleotides are shown in green. LNAs are indicated with a plus in front of corresponding nucleotide letter; LNA nucleotides are written in brackets.

* Predicted T_m values are for all duplexes with no crowders. ** The difference between experimental and predicted ΔT_m.

We measured the calibration curve for ct DNA:Eva Green complex (Supplementary Figure 10A). Samples were prepared in duplicate, by mixing a known amount of ct DNA with 6 µL 20X stock of Eva Green dye (Biotium) in 1X PBS buffer, pH 7.2, and measuring fluorescence right away. Total sample volume was kept at 100 µL. Final concentrations of ct DNA per sample were as follows: 5 nM, 1 nM, 500 pM, 250 pM, 100 pM, 50 pM, 25 pM, 10 pM, 5 pM, 1 pM. The read out was obtained using 384-well plate Roche Light Cycler 480 plate reader. The data was analyzed using Light Cycler fluorescence analysis software following the manufacturer's recommendations. The deviation between two independent measurements was below 2%. Resulting curve is given below.

Supplementary Figure 10. Calibration curve for ct DNA:Eva Green complex (A), and chemical structure of biotin modification incorporated into capture oligonucleotides (B).

A)

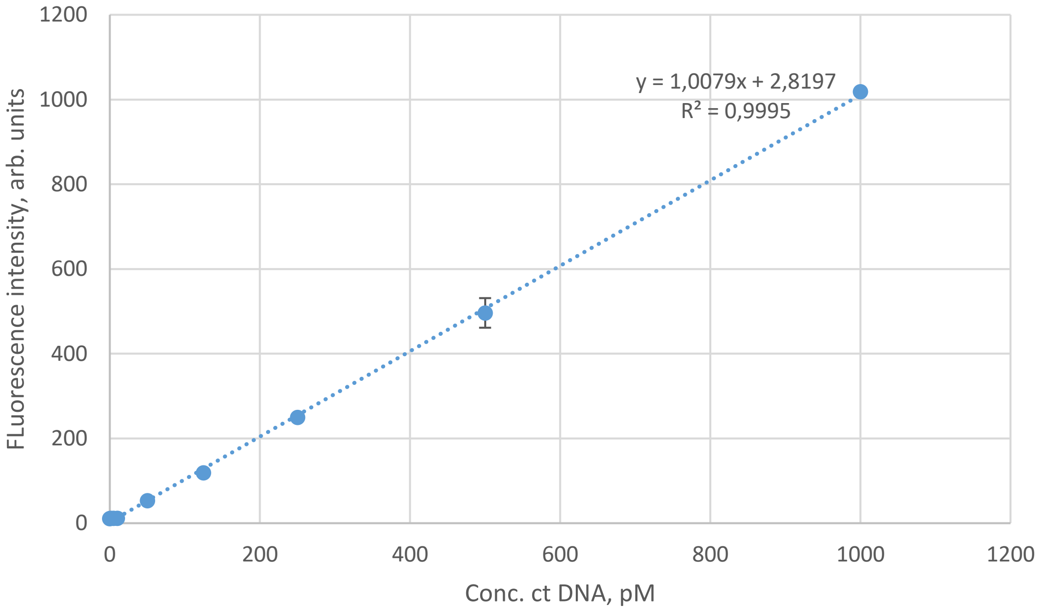

B)

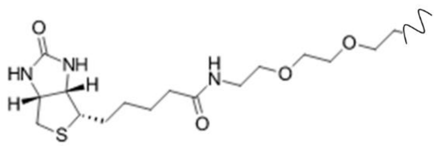

We performed control bead-bait hybridization studies for *BRAF* and *EGFR* targets, as described in Methods. For this control, we were using capture probes DR1-bt (5'-biotin-TAG CTA CAG AGA AAT CTC GAT), and DR3-bt (5’-biotin- ACT GTA CAT GAG AAA CTT TTT CTC). For chemical structure of biotin modification see Supplementary Figure 10B. Concentrations of RNA by bead-bait assay were calculated using the calibration curve given in Figure 10. Concentrations of RNA by RT-qPCR were determined by internal control.

Supplementary Table 22. Resulting RNA concentrations determined by bead-bait assay and LNA RT-qPCR for *BRAF* and *EGFR* control oncogenes.[1]

	**Bead-bait assay, Results for replicates**			Average Conc, pM	CV,%
DR1-bt/BRAF V600E	321	305	318	314,7	2,2
DR3-bt/EGFR L858R	121	124	122	122,3	1,0
	**LNA PCR**				
Target	Concentration, replicates			Average Conc, pM	CV,%
BRAF V600E	310	332	302	314,7	4,0
EGFR L858R	116	127	122	121,7	3,7

We proceeded with bead-bait hybridization and PCR detection of *KRAS* targets, following procedures described in Methods. Bait probe sequences used in the assay are given in Supplementary Table 23. For the prediction, they were used without any modification. However, in the bead-bait assay they were used as 5’ biotinylated oligonucleotides.
RNA extracted from MRC-5 cell line has been used as a negative control, whereas HT-29 RNA contained *KRAS* mutation.

The key results of *KRAS* detection are shown in Supplementary Table 23. In the Table, data is arranges with best performing bait probes in the upper part of the table (ΔT_m match-mismatch above 5.2 °C); and control probes with lower mismatch discrimination: DR5-M1199, DR5-M486, DR19-M641 and DR21-M729.
RT-qPCR has been performed using same set of primers, given in Methods. Therefore only one value has been obtained for RT-qPCR per target RNA.

Key results that prove better performance of rationally designed bait probes (DR26-M283, DR26-M208, DR23-M208, DR23-M283, DR29-M97 and DR29-M17), vs. RT-qPCR, is indicated in bold. We observe higher concentration of target RNA determined by bead-bait assay vs. RT-qPCR, and lower concentration of negative control.

Supplementary Table 23. Bead-bait assay results and LNA RT-qPCR for *KRAS* G12D.[1]

	Bead-bait assay, results for replicates			Average conc, pM	CV,%
**Target: HT29 DNA (mutated)**					
**Capture probe #**					
DR26-M283	113	116	111	113,33	1,81
DR26-M208	111	102	104	105,67	3,65
DR23-208	104	106	102	104,00	1,57
DR23-M283	122	122	114	119,33	3,16
DR29-M97	113	112	106	110,33	2,80
DR29-M17	121	122	117	120,00	1,80
DR5-M1199	144	140	142	142,00	1,15
DR5-M486	155	165	156	158,67	2,83
DR19-M641	160	162	163	161,67	0,77
DR21-M729	170	164	165	166,33	1,58
**Target: MRC5 DNA (wild type)**					
**Capture probe #**					
DR26-M283	6	7	7	6,67	7,07
DR26-M208	9	9	7	8,33	11,31
DR23-208	7	8	8	7,67	6,15
DR23-M283	6	6	7	6,33	7,44
DR29-M97	9	9	8	8,67	5,44
DR29-M17	7	7	6	6,67	7,07
DR5-M1199	5	5	6	5,33	8,84
DR5-M486	6	7	9	7,33	17,01
DR19-M641	5	5	6	5,33	8,84
DR21-M729	7	8	8	7,67	6,15
	Concentration data for replicates **LNA PCR**			Average conc, pM	CV,%
**HT29 target**	20	24	17	20,33	14,10
**MRC5 target**	22	21	27	23,33	11,25

Target dilution study was carried out similarly to the bead-bait assay described in Methods, using a total of 100 ng RNA per analysis and capture probes DR26-M283, DR26-208 and DR29-17. RT-qPCR has been carried out for the same RNA samples as well, using primers for RT and qPCR given above for *KRAS* target.

RNA samples were prepared by mixing wild type RNA (obtained from MRC-56 cell line), and *KRAS* mutant RNA (obtained from HT-29 cell line), as follows: 100% mutant, 50% mutant, 25% mutant, 12.5 % mutant, 6.25 % mutant, 3.125 % mutant, 1.5626 % mutant and 100% wild type RNA. Each sample was an alysed in triplicate in three independent experiments. The obtained data is shown in Supplementary Table 24 and Supplementary Figures 11&12.

Limit of detection (LOD) was determined as the minimal amount of mutant RNA in the sample with fluorescence response over 3-fold higher than signal for 100% wild type sample [Suppl. Ref.14]. For RT-qPCR, LOD has been calculated as minimal amount of mutant RNA in the sample with Ct response over 3-cycle higher than Ct value for 100% wild type sample [Suppl. Ref. 15].

Supplementary Table 24. Concentration values for target dilution assay - data for independent triplicate measurements, using selected capture probes.

Sample	**Conc., pM/ Probe # #DR26-M283**			CV,%	**Conc., pM/ Probe # #DR26-M208**			CV,%	**Conc., pM/ Probe # #DR29-M17**			CV,%
100% mutant	1001,00	1000,00	1021,00	0,96	998,00	990,00	985,00	0,54	1023,00	1011,00	1009,00	0,61
50 % mutant	677,00	670,00	665,00	0,73	665,00	664,00	679,00	1,02	687,00	680,00	688,00	0,52
25 % mutant	477,00	400,00	422,00	7,48	421,00	443,00	455,00	3,20	422,00	400,00	422,00	2,50
12.5 % mutant	244,00	223,00	231,00	3,72	221,00	243,00	243,00	4,40	225,00	226,00	220,00	1,17
6.25 % mutant	123,00	122,00	120,00	1,03	120,00	126,00	121,00	2,15	121,00	126,00	120,00	2,15
3.125 % mutant	43,00	45,00	40,00	4,82	44,00	47,00	48,00	3,67	47,00	50,00	55,00	6,51
1.5625 % mutant	22,00	22,00	22,00	0,00	21,00	23,00	22,00	3,71	26,00	25,00	22,00	6,98
100 % wild-type	11,00	11,00	12,00	4,16	12,00	12,00	12,00	0,00	11,00	11,00	12,00	4,16
CV average, %				2,86				2,34				3,08

Supplementary Table 25. Average values for triplicate results from independent measurements; bead-bait target dilution assay

Sample	#DR26-M283	#DR26-M208	#DR29-M17
100% mutant	1001,0	998,0	1023,0
50 % mutant	677,0	665,0	687,0
25 % mutant	477,0	421,0	422,0
12.5 % mutant	244,0	221,0	225,0
6.25 % mutant	123,0	120,0	121,0
3.125 % mutant	43,0	44,0	47,0
1.5625 % mutant	22,0	21,0	26,0
100 % wild-type	11,0	12,0	12,0

Supplementary Table 26. Bead-bait assay results for *KRAS* G12D – target dilution assay.

Capture probe #	Bait probe sequence, 5'→3'	Predicted ΔT_m, °C mutant – wild type	LOD: Bead assay, % mutant RNA/average CV, %
**#DR26-M283**	5'-bt-G{+A}TTGCG{+T}AGGCAAGAG{+T}GC	7.33	2.4/2.8
**#DR26-M208**	5'-bt-G{+A}{+T}TGCGTAGGCAAGAG{+T}GC	7.07	2.6/2.3
**#DR29-M17**	5'-bt-{+A}{+T}TGCGTAGGCAAGAG{+T}GCC	5.23	2.4.8/3.1

LNAs are indicated with a plus in front of corresponding nucleotide letter; LNA nucleotides are written in brackets.

Supplementary Table 27. PCR data for target dilution assay, triplicate independent measurements and average Ct values.

Sample:	Ct result, run #				
	1st	2nd	3rd	CV,%	**Ct, average**
100% mutant	24	23,2	27	6,61	24,73
50 % mutant	26	26,8	27,2	1,87	26,67
25 % mutant	27,2	28	27,2	1,37	27,47
12.5 % mutant	28,5	29	28	1,43	28,50
6.25 % mutant	29,9	30	29,4	0,88	29,77
3.125 % mutant	31	31,2	31	0,30	31,07
1.5625 % mutant	31,5	28,2	32	5,52	30,57
100 % wild-type	32,0	32	32,5	0,73	32,17
**Average CV, %**				**2,3**	

LOD: RT-qPCR, % mutant RNA /average CV, % : 12.5%/2.3.

Supplementary Figure 11. Target dilution study for bead-bait (A) and RT-qPCR assays (B).

A)

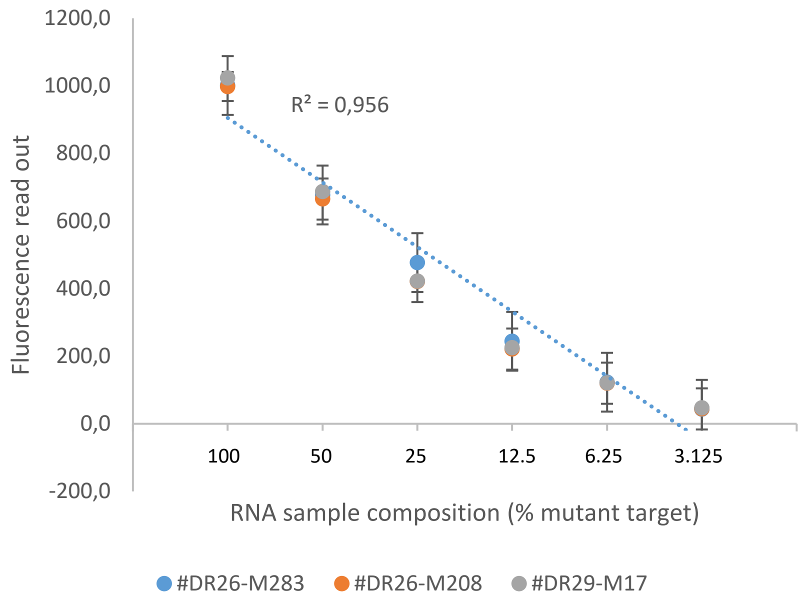

B)

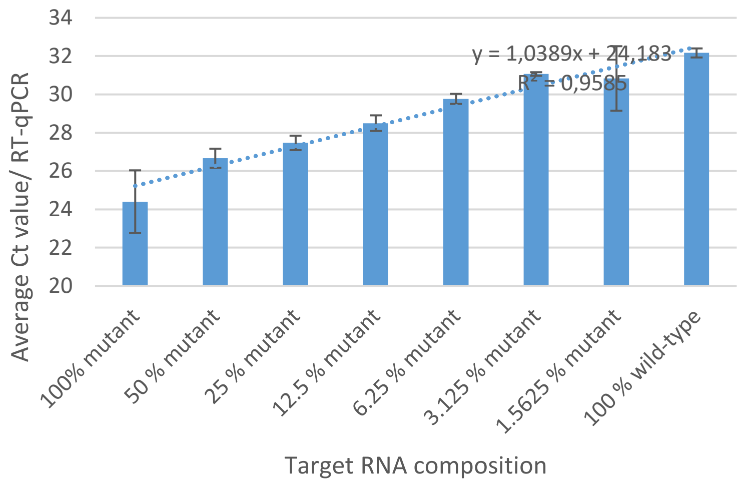

Average data points are given for each sample composition. Error has been estimated for the three independent measurements. Target dilution study was carried out using a total of 100 ng RNA per analysis and capture probes DR26-M283, DR26-208 and DR29-17. RT-qPCR has been carried out for the same RNA samples as well, using primers for RT and qPCR given above for *KRAS* target. RNA samples were prepared by mixing wild type RNA (obtained from MRC-56 cell line), and *KRAS* mutant RNA (obtained from HT-29 cell line), in a desired ratio of mutant to wild-type.
