# Supplementary material for: Optical and theoretical study of strand recognition by nucleic acid probes
Source: Commun Chem. 2020 Aug 11;3:111. doi: 10.1038/s42004-020-00362-5 (PMC9814704; doi:10.1038/s42004-020-00362-5)
Supplement: Supplementary file 2 — Description of Additional Supplementary Files [file 42004_2020_362_MOESM2_ESM.pdf]

Description of Additional Supplementary Files

File Name: Supplementary Data 1
Description: **Computer design of oligonucleotide probes for KRAS G12D oncogene.** The data file 1 shows designs and theoretical prediction of T_m for various probe designs targeting *KRAS* G12D RNA; data given for mutant (full match) and wild-type (mismatch) target
